# Enhancing product quality incorporating additive manufacturing on recycled inventory system with deterioration and delayed payment

**DOI:** 10.1038/s41598-026-48194-y

**Published:** 2026-04-21

**Authors:** B. Priskilla, G. S. Mahapatra, M. V. A. Raju Bahubalendruni, Dhinesh Balasubramanian, Utku Kale, Artruras Kilikevičius

**Affiliations:** 1 Department of Mathematics, National Institute of Technology Puducherry, Karaikal, India; 2 Department of Mechanical Engineering, National Institute of Technology Puducherry, Karaikal, India; 3 Department of Port Engineering, Lithuanian Maritime Academy (LMA) Vilnius Gediminas Technical University, Klaipeda, Lithuania; 4 Department of Aeronautics and Naval Architecture, Faculty of Transportation Engineering and Vehicle Engineering, Budapest University of Technology and Economics, Műegyetem rkp. 3., Budapest, H-1111 Hungary; 5 Mechanical Science Institute, Vilnius Gediminas Technical University, Plytinės g. 25, LT-10105 Vilnius, Lithuania

**Keywords:** Quality, Delay payment, 3D-printing technology, Sustainable, Shortages, Engineering, Environmental sciences, Environmental social sciences, Mathematics and computing

## Abstract

This study develops an environmentally sustainable inventory system for retailers engaged in the sale of recycled products derived from the reprocessing industry. The model integrates an investment framework in additive manufacturing technology aimed at minimizing emissions and waste during production while simultaneously enhancing product quality. The primary objective of the proposed inventory system is to elevate the quality standards of recycled products and improve overall profitability. Furthermore, the model incorporates demand-dependent reliability, pricing, and quality factors to maximize profit while satisfying customer demand. It also considers deteriorating items, potential shortages, and trade credit policies to ensure a realistic operational structure. The applicability and efficiency of the proposed approach have been demonstrated through a case study involving an existing retailer, confirming its capability to enhance profitability while promoting eco-friendly production via additive manufacturing (AM). The results indicated that the integration of AM technology led to more than 2% improvement in total profit for the inventory system. In addition, a sensitivity analysis was performed to assess the influence of key parameters on the optimal solution.

## Introduction

As the world grapples with the mounting issue of plastic waste, innovative approaches are essential to mitigate the environmental impact of plastic products. One such approach is to leverage additive manufacturing (AM) or 3D printing technology to create innovative and high-quality products. Use of AM not only reduces the need for new production but also supports the principles of the circular economy and reduces environmental waste. This offers precise control over material properties and product design, enabling the creation of customized and high-performance products made from recycled materials over traditional methods. Ashok et al. analysed various AM technologies that offer a significant advantage in reducing product development timelines for intricate structures^1^. Furthermore, researchers discussed layer-by-layer additive process ensures high precision, enhancing manufacturing quality and reliability^2,3^ and design an optimized, sustainable reverse logistics model for PVC recycling that minimizes costs and environmental impacts while improving operational efficiency. As AM integrates into manufacturing, its efficiency, sustainability, and adaptability reshape industries, promising a future marked by reduced waste, optimized inventory, and elevated production standards.

In the inventory system, one of the important parameters known as demand must be considered when making decisions about the stock and output of the warehouses. Researchers are emphasizing pivotal factors in inventory systems, notably focusing on aspects such as degradation, demand dynamics, and the assurance of reliability and quality. One of the significant challenges confronting the community today is the recycling of plastics by developing innovative and sustainable recycling with minimal environmental impact. In most of the research, facts are needed to answer the specific queries on inventory regarding AM as follows:

- Need to answer approaches to improve the quality of recycled products using additive manufacturing technology.
- How can a sustainable recycled inventory system be created through the use of AM technology?
- Analysis on investment from retailers in 3D printers can support the production of recycled products.
- How can the reliability of recycled plastic products be ensured while maximizing overall profit?

Furthermore, this advancement has motivated retailers to invest in 3D printing technologies, fostering the broader adoption of AM for sustainable and efficient inventory systems.

## Literature review

Nowadays, the majority of buyers prefer the acquisition of high-quality and dependable. Different scenarios in production inventory models have been analysed that focus on particular situations incorporating demand influenced by both price and stock levels, decay of products^4^, partial backlog^5^, stochastic demand^6^, and product reliability^7^. Researchers^8–10^ explored preventive maintenance strategies for manufacturing systems deteriorating in reliability and quality, assuming constant demand or the demand rates that vary with both stock levels and time^11^. Research has been developed on an inventory framework that incorporates fuzzy demand subject to price discounts^12^ .

The degradation rate significantly influences the optimal policy^13,14^. Plastics degrade over time due to factors such as temperature, humidity, light exposure, and chemical interactions with other substances. Many researchers have been studying inventory models for decaying items in recent years on the smart manufacturing system of degrading products through the application of preservation technologies. Numerous researchers have created inventory models for perishable items with varying decay rates instead of a steady deterioration rate^6,15^.

In industry, payment delay refers to the time it takes a company to settle a bill after receiving it, whereas late payments refer to payments that are delayed beyond the agreed-upon date. Several findings suggest that policies allowing for payment delays can result in heightened demand^16^, whereas prepayment policies tend to diminish demand, depending on the duration of the prepayment terms^17,18^.

Inventory shortages occur when demand exceeds available stock, leading to lost sales, reduced customer satisfaction, and reputational risks. To mitigate these challenges, advanced inventory management uses data-driven strategies like demand forecasting, safety stock optimization, and real-time tracking to minimize stockouts and improve efficiency. San-Jose et al. created a sustainable inventory model with complete backlogs. developed a bi-objective model to make supply chains sustainable by minimizing production waste and reducing environmental emissions^19^.

The implementation of AM technologies in inventory models for enhancing defect- free production, life cycle, and cost-controlled inventory reduces the environmental impact irrespective of the kind of material and production process^20^. Simon et al. created a life cycle inventory framework for stereolithography, a commonly employed AM method^21^. Roux et al. developed an environmental evaluation of an AM method that used a generic framework to examine the environmental effect of 3D concrete printing via a parametric model^22^. Roozkhosh et al. developed a bi-objective sustainable supply chain for reduce waste generation and pollution through the integration of AM^23^. Hafiz et al. reviewed the use of recycled polymers in 3D printing, emphasizing recycling methods, reinforcements, and parameter optimization for sustainable production^24^. AM technology has been designed for strategies in supply chain network^25^, conventional warehouse inventory system^26^, and also on the inventory model^27^ so that businesses may enhance. Researchers investigated and compared the effects of the incorporation of AM technology into the supply chain networks^28–32^.

### Research aperture on the topic

This study further contrasts traditional manufacturing methods with AM technology within the inventory framework. There is a notable gap in addressing the integrated sustainable inventory model formulated to enhance the quality and reliability of recycled products using AM technology. This innovative model incorporates realistic factors such as price, time, quality, reliability demand, delayed payment, AM technology investment, etc., aiming to achieve sustainable development. This work aims to address the stated research gap by pursuing the following objectives:

- To develop a sustainable EOQ model integrating AM (3D printing) for ecofriendly inventory management.
- To enhances product quality and reliability while utilizing recycled plastic materials in production.
- To evaluate the economic and environmental benefits of adopting 3D printing technology in retailer operations.

Table 1 highlights the research gap and emphasises that this article is a pioneering effort to address all of the factors discussed collectively. More specific contributions to this manuscript are as follows:

- Introduce the additive manufacturing technology investments to increase the quality, reliability, and profitability of recycled products.
- A sustainable inventory model using AM technology for emission reduction and waste minimization to enhance the quality of recycled plastic products.

**Table 1:** Contribution-based comparative analysis of this work with earlier models.

Reference	Sustainable	EOQ	Price dependent demand	Time dependent demand	Quality dependent demand	Reliability dependent demand	AM Technology or 3D printing technology	Delay payment
^33^	X	X	X	X	X	X	✓	X
^34^	X	✓	✓	X	X	X	X	X
^35^	X	✓	✓	X	X	X	X	X
^36^	X	X	✓	X	X	X	X	X
^37^	✓	X	X	X	X	X	✓	X
^38^	X	✓	✓	✓	X	X	X	X
^39^	✓			X	X	X	✓	X
^40^	X	✓	✓	X	X	X	X	X
^41^	X	X	X	X	X	X	✓	X
^42^	✓	✓	✓	X	X	X	X	✓
^12^	✓	✓	✓	X	X	X	X	X
^43^	✓	X	X	X	X	X	✓	X
^44^	✓	X	✓	X	X	X	X	X
^45^	X	✓	✓	✓	X	X	X	✓
^46^	✓	✓	X	X	X	✓	✓	X
^47^	X	✓	✓	X	X	X	X	✓
^48^	✓	✓	X	✓	X	X	X	✓
^49^	X	X	X	X	X	X	✓	X
^50^	X	X	X	✓	X	X	✓	X
^51^	✓	X	✓	X	X	X	X	X
^52^	X	✓	✓	X	X	X	X	X
This paper	✓	✓	✓	✓	✓	✓	✓	✓

## Development of sustainable inventory system

In the proposed analytical framework, AM technology is introduced within the context of a retailer-oriented EOQ system, where inventory policies are primarily influenced by market demand characteristics and the perceived quality of products rather than by upstream production constraints. Where inventory decisions are primarily influenced by customer demand and perceived product quality rather than upstream production factors. Investment in AM is therefore interpreted as a technological intervention that enhances the structural integrity, reliability, and consistency of recycled plastic products. The improved process precision reduces manufacturing defects and strengthens functional performance. As product reliability increases, consumer confidence and market acceptance of recycled plastics expand, leading to higher demand. Retailers consequently revise replenishment cycles and order quantities accordingly, linking AM-driven quality improvement directly with optimal inventory decisions and market sustainability. This study is consistent with a negligible replenishment rate of the horizon for inventory planning that extends infinitely. The following considerations are essential and consistent with the review of this study.

- The demand rate for this model which depends on the price, reliability and quality and increases with time i.e., $\mathrm{R}\left(p,r,q\right)=\mathrm{a}+\left(xr+yq-bp\right)\mathrm{t},0\le t\le {t}_{i}$, here a is initial demand.
- Degradation initiates immediately upon lot reception, and its rate remains constant at deterioration rate denoted by $\uptheta \left(0<\uptheta <1\right)$.
- If the retailer settles the payment within the designated credit period D provided by the supplier, they will avoid any interest fees. Conversely, if the retailer makes the payment after the credit term D, they will incur interest at the rate *I*_*p*_.
- Case I $(D<{t}_{1})$: During the time interval from the start of the cycle to the end of the credit period D the retailer is not required to make any payment to the supplier and can earn interest on the sales revenue at an annual rate *I*_*e*_. However, when the credit period expires, some inventory still remains in stock. Consequently, the retailer must finance the remaining inventory and pay interest at a rate *I*_*p*_ until the inventory is completely depleted at time *t*_*1*_.
- Case II $(D\ge {t}_{1})$: The supplier provides a credit period longer than the retailer’s inventory cycle. In this case, the retailer is able to sell the entire inventory before payment becomes due, eliminating the need for financing costs and potentially allowing the retailer to earn interest on the revenue generated from sales.
- The rate at which this model is in demand relies on product reliability, expressed as $\mathrm{r}\left(s\right)=\left(1-{e}^{-{a}_{0}s}\right)$. The reliability of products offered by the retailer is influenced by the quality of goods provided by the manufacturer^53^.
- Quality of items in the inventory system is $q\left(A\right)=\omega \left(\frac{eA}{1+eA}\right)$ i.e. $q\%$ of quality item’s present in the inventory. Here u is the amount of recycling products when 3D technology is investmented, and e is the efficiency of 3D technology in recycling products. The quality $q=0$, when $A=0$, and tends to u when $A\to \infty$. The cost function of investment, $q(A)$ is possesses continuous derivatives $q^{\prime } \left( A \right) > 0,q^{\prime \prime } \left( A \right) < 0$^54^.

**Fig. 1:**
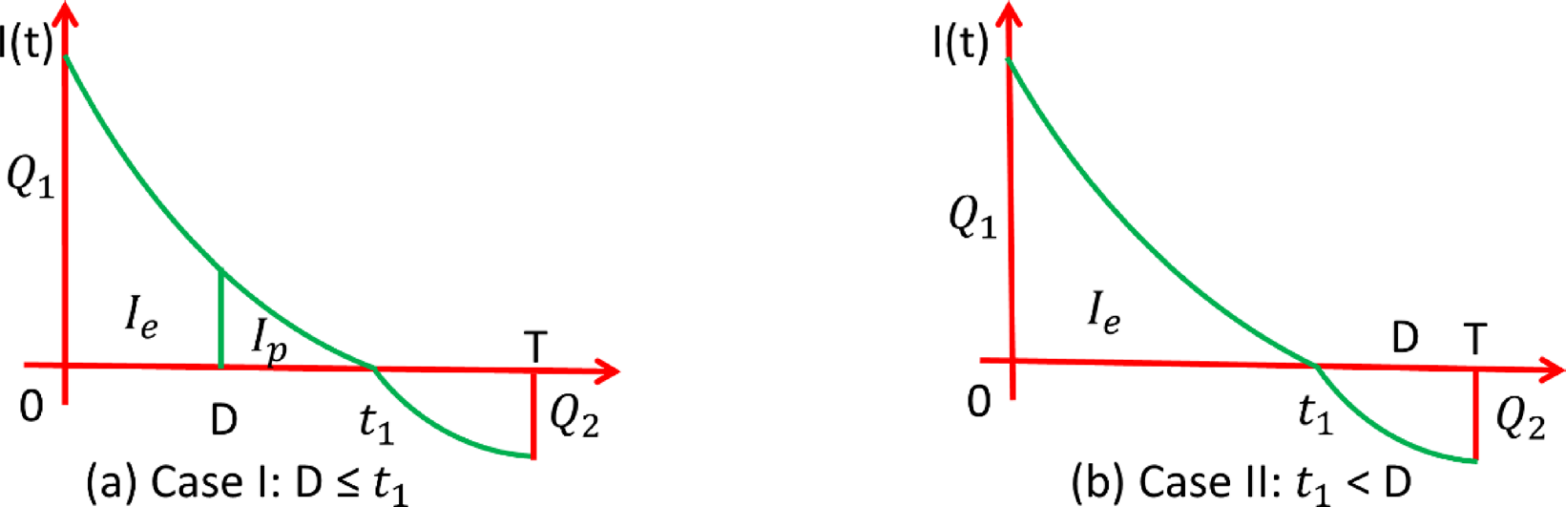
Visual depiction of the inventory model with two sub periods system.

Let ${Q}_{1}$ is the starting point for the inventory level. As a result of demand and deterioration, the inventory level drops. Figure 1 shows two subperiods of *T*, when the inventory level drops to zero at time t1, there are shortages in the fully backlogged period $\left({t}_{1}, \mathrm{T}\right).$

1 $$\frac{dI\left(t\right)}{dt}+\theta I\left(t\right)=-R\left(p,r,q\right), 0\le t\le {t}_{1}.$$

2 $$\frac{dI\left(t\right)}{dt}=-R\left(p,r,q\right), {t}_{1}\le t\le T$$

with the condition $I\left(0\right)={Q}_{1},I\left({t}_{1}\right)=0,I\left(T\right)=-{Q}_{2}$ and $I(t)$ is continuous at $t={t}_{1}$

Resolving Eq. (1) utilizing the provided condition $I({t}_{1}) = 0$, one has

3 $$I\left(t\right)=\frac{1}{{\theta }^{2}}\left(\left({e}^{\theta \left({t}_{1}-t\right)}-1\right)\left(a\theta -\left(xr+yq-bp\right)\right)+\theta \left(-bp\right)\left({e}^{\theta \left({t}_{1}-t\right)}{t}_{1}-t\right)\right)$$

$$where\;0\le t\le {t}_{1}$$

Once more, leveraging the boundary condition $I(0) = {Q}_{1}$, we can find

4 $${Q}_{1}=\frac{1}{{\theta }^{2}}\left(\left({e}^{\theta {t}_{1}}-1\right)\left(a\theta -\left(xr+yq -bp\right)\right)+\theta {t}_{1}\left(xr+yq -bp\right){e}^{\theta {t}_{1}}\right)$$

Addressing the Eq. (2) under the specified condition $I({t}_{1})=0$, yields

5 $$I\left(t\right)=a\left({t}_{1}-t\right)+\frac{\left(xr+yq-bp\right)}{2}\left({t}_{1}^{2}-{t}^{2}\right), where {t}_{1}\le t\le T$$

once again, utilizing the auxiliary condition $I(T)=-{Q}_{2}$, one can derive

6 $${Q}_{2}=a\left(T-{t}_{1}\right)+\frac{\left(xr+yq-bp\right)}{2}\left({T}^{2}-{t}_{1}^{2}\right)$$

7 $$Q=\frac{1}{\theta { }^{2}}\left(\left({e}^{\theta {t}_{1}}-1\right)\left(a\theta -\left(xr+yq -bp\right)\right)+\theta {t}_{1}\left(xr+yq -bp\right){e}^{\theta {t}_{1}}\right) +\left(a\left(T-{t}_{1}\right)+\frac{\left(xr+yq -bp\right)}{2}\left({T}^{2}-{t}_{1}^{2}\right)\right)$$

## Derivation of costs of inventory system

The cost interpretation of a business framework is vital to deciding the system’s endeavour for an inventory model with a supplier or manufacturer.

Ordering cost (${C}_{o}$): A constant expense associated with processing each order cycle, as given by

8 $$OC={C}_{o}$$

Holding cost (*C*_*h*_ unit per unit time): It refers to the costs or losses incurred by a business for maintaining inventory over a specified duration. Using expressions for $I(t)$ appropriately by referring to Eq. (3), the cost of holding $HC={C}_{h}{\int }_{0}^{{t}_{1}}I\left(t\right)dt$ for the sellable inventory is expressible as:

9 $$HC=\frac{{C}_{h}}{{\theta }^{3}}\left(\left({e}^{\theta {t}_{1}} -1\right)\left(a\theta -\left(xr+yq-bp\right)\right)-\theta {t}_{1}\left(a\theta -\left(xr+yq-bp\right){e}^{\theta {t}_{1}}\right)-\frac{{\theta }^{2}{t}_{1}^{2}}{2}\left(xr+yq-bp\right)\right)$$

An organization’s purchasing cost (${C}_{p}$ per unit time) is the amount it spends on purchasing the products and services it needs to operate or produce a product.

Purchasing cost (*PC*): The total cost expended by the merchant throughout the whole planning cycle for acquiring the requisite quantity of product in the form of purchase amounts is

10 $$PC=\frac{{C}_{p}}{{\theta }^{2}}\left({e}^{\uptheta {t}_{1}}-1\right)\left(a\uptheta -xr-yq+bp\right)+{t}_{1}\uptheta \left(xr+yq-bp\right){e}^{\uptheta {t}_{1}}+{\uptheta }^{2}a\left(T-{t}_{1}\right)+{\uptheta }^{2}\frac{\left(xr+yq-bp\right)}{2}\left({T}^{2}-{t}_{1}^{2}\right)$$

The shortage cost (${C}_{b}$) represents the expenditures or losses incurred by an organization due to insufficient inventory or product availability for customer demand, resulting in missed sales opportunities or customer dissatisfaction, which is evaluated as:

11 $$\begin{aligned} SC = & - C_{b} \int_{{(t_{1} )}}^{T} {I(t)dt} \\ = & - C_{b} (at_{1} (T - t_{1} ) - a/2(T^{2} - t_{1}^{2} ) \\ & + (xr + yq - bp)/6(3t_{1}^{2} (T - t_{1} ) - (T^{3} - t_{1}^{3} ))) \\ \end{aligned}$$

Sales revenue: The total amount of money that the inventory system will make from the sale of goods throughout the planning cycle is provided by

12 $$SR=p\left({\int }_{0}^{{t}_{1}}R\left(p,r,q\right)dt+{Q}_{2}\right)=p\left(aT+\frac{\left(xr+yq-bp\right)}{2}{T}^{2}\right)$$

Deterioration cost (*C*_*d*_ of deterioration per unit over time): From the stock that the retailer receives in excellent condition, a certain percentage of the items begin to deteriorate over time; as a result, the cost of deterioration is calculated as:

13 $$\begin{aligned} DC =  & C_{d} \left({Q_{1} - \int_{0}^{{t_{1}}} {R\left( {p,r,q} \right)dt} } \right) \\ & = \frac{C_{d}}{\theta ^{2}}{\left[{\left({e^{{\theta t_{1}}}-1}\right)(a\theta-(xr+yq-bp)+\theta t_{1}\left({xr + yq - bp} \right)e^{{\theta t_{1}}}} \right)}\left.{- \theta^{2}\left({at_{1}+ \frac{{\left({xr + yq - bp}\right)t_{1}^{2}}}{2}}\right)}\right]\\ \end{aligned}$$

The AM technology investment cost per cycle is given by

14 $$AIC=AT$$

**Delay based payment frameworks**

There are two possible results from this inventory system: either the buyer’s conditional payment delays are larger or shorter than the cycle time. In order to determine the overall inventory costs, we evaluate the interest paid in both situations due to payment delays.

**Case I** **:** $({\boldsymbol{D}}<{{\boldsymbol{t}}}_{1})$ **payment delay is less than the cycle time**

The interest earned from 0 to ${t}_{1}$ is provided by

15 $$I{E}_{1}={C}_{p}{I}_{e}{\int }_{0}^{{t}_{1}}tR\left(p,r,q\right)dt={C}_{p}{I}_{e}\left(\frac{a{t}_{1}^{2}}{2}+\left(xr+yq-bp\right)\frac{{t}_{1}^{3}}{3}\right)$$

The retailer’s cycle-specific interest rate is calculated by

16 $$\begin{aligned} IP_{1} = & C_{p} I_{p} \int_{D}^{T} {I(t)dt} = C_{p} I_{p} \left( {\int_{D}^{{(t_{1} )}} {I(t)dt} + \int_{{(t_{1} )}}^{T} {I(t)dt} } \right) \\ = & \frac{{(C_{p} I_{p} )}}{{\theta ^{3} }}\bigg[((e^{\theta (t_{1} - D)} - 1)(a\theta - (xr + yq - bp)) \\ & + \theta (xr + yq - bp)t_{1} ) - \theta (a\theta - (xs + yq - bp))(t_{1} - D) \\ & - \frac{{(\theta ^{2} (xr + yq - bp))}}{2}(t_{1}^{2} - D^{2} ) + \left. { + \frac{{\theta ^{3} \left( {T - t_{1} } \right)}}{6}\left( {3a\left( {t_{1} - T} \right) + \left( {xr + yq - bp} \right)\left( {2t_{1}^{2} - T^{2} - Tt_{1} } \right)} \right)}\right] \\ \end{aligned}$$

In case $(D<{t}_{1})$, the total inventory cost per unit of time $T{P}_{1}\left({t}_{1},T\right)$ is calculated as:

$$\frac{1}{\mathrm{T}}\left(\mathrm{SR}-\mathrm{HC}-\mathrm{PC}-\mathrm{OC}-\mathrm{DC}-\mathrm{SC}-{\mathrm{IP}}_{1}+ {\mathrm{IE}}_{1}-\mathrm{AIC}\right)$$

17 $$\begin{aligned} TP_{1} \left( {t_{1} ,T} \right) = & \frac{1}{T}\left[ {p\left( {aT + \frac{{\left( {xr + yq - bp} \right)}}{2}T^{2} } \right)} \right. \\ & - \frac{{C_{h} }}{{\theta ^{3} }}\left( {\left( {e^{{\theta t_{1} }} - 1} \right)(a\theta - (xr + yq - bp)} \right) \\ & - \theta t_{1} \left( {a\theta - \left( {xr + yq - bp} \right)e^{{\theta t_{1} }} } \right) - \frac{{\theta ^{2} t_{1}^{2} }}{2}(xr + yq - bp)) \\ & - \frac{{C_{p} }}{{\theta ^{2} }}\left( {\left( {e^{{\theta t_{1} }} - 1} \right)\left( {a\theta - \left( {xr + yq - bp} \right)} \right)} \right. \\ & + t_{1} \theta \left( {xr + yq - bp} \right)e^{{\theta t_{1} }} + a\theta ^{2} \left( {T - t_{1} } \right) \\ & \left. { + \frac{{\theta ^{2} \left( {xr + yq - bp} \right)}}{2}\left( {T^{2} - t_{1}^{2} } \right)} \right) - C_{0} \\ & - \frac{{C_{d} }}{{\theta ^{2} }}\left( {\left( {e^{{\theta t_{1} }} - 1} \right)\left( {a\theta - \left( {xr + yq - bp} \right)} \right)} \right. \\ & \left. { - \theta t_{1} \left( {xr + yq - bp} \right)e^{{\theta t_{1} }} - \theta ^{2} \left( {at_{1} + \frac{{\left( {xr + yq - bp} \right)t_{1}^{2} }}{2}} \right)} \right) \\ & + C_{b} \left( {at_{1} \left( {T - t_{1} } \right) - \frac{a}{2}\left( {T^{2} - t_{1}^{2} } \right) + \frac{{\left( {xr + yq - bp} \right)}}{6}\left( {3t_{1}^{2} \left( {T - t_{1} } \right) - \left( {T^{3} - t_{1}^{3} } \right)} \right)} \right) \\ & - \frac{{C_{p} I_{p} }}{{\theta ^{3} }}\left( {\left( {e^{{\left( {t_{1} - D} \right)}} - 1} \right)\left( {a\theta - \left( {xr + yq - bp} \right)} \right) + \theta \left( {xr + yq - bp} \right)t_{1} } \right. \\ & - \theta \left( {a\theta - \left( {xr + yq - bp} \right)} \right)\left( {t_{1} - D} \right) - \frac{{\theta ^{2} \left( {xr + yq - bp} \right)}}{2}\left( {t_{1}^{2} - D^{2} } \right) \\ & \left. { + \frac{{\theta ^{3} \left( {T - t_{1} } \right)}}{6}\left( {3a\left( {t_{1} - T} \right) + \left( {xr + yq - bp} \right)\left( {2t_{1}^{2} - T^{2} - Tt_{1} } \right)} \right)} \right) \\ & + C_{p} I_{e} \left( {\frac{{at_{1}^{2} }}{2} + \left( {xr + yq - bp} \right)\frac{{t_{1}^{3} }}{3}} \right) - AT\bigg] \\ \end{aligned}$$

**Case II:** $({{\boldsymbol{t}}}_{1}<{\boldsymbol{D}})$, **payment delay is greater than the cycle time:**

Here Ie is the retailer interest earned per cycle at the return rate. If ${t}_{1}<D$, per cycle’s annual interest is given by

18 $$I{E}_{2}={C}_{p}{I}_{e}\left({\int }_{0}^{{t}_{1}}tR\left(p,r,q\right)dt+\left(D-{t}_{1}\right){\int }_{0}^{{t}_{1}}R\left(p,r,q\right)dt\right) ={C}_{p}{I}_{e}\left(3a{t}_{1}\left(2D-{t}_{1}\right)+\left(xr+yq-bp\right){t}_{1}^{2}\left(3D-{t}_{1}\right)\right)$$

In case $(D>{t}_{1})$, the total inventory cost per unit of time $T{P}_{2}\left({t}_{1},T\right)$ is calculated as:

$$\frac{1}{T}\left(SR-HC-PC-OC-DC-SC-I{P}_{2}+ I{E}_{2}-AIC\right)$$

19 $$\begin{aligned} {\mathrm{TP}}_{2} \left( {t_{1} ,T} \right) = & \frac{1}{T}\left[ {p\left( {aT + \frac{{\left( {xr + yq - bp} \right)}}{2}T^{2} } \right) - \frac{{C_{h} }}{{\theta ^{3} }}\left( {\left( {e^{{\theta t_{1} }} - 1} \right)(a\theta - (xr + yq - bp)} \right)} \right. \\ & - \theta t_{1} \left( {a\theta - \left( {xr + yq - bp} \right)e^{{\theta t_{1} }} } \right) - \frac{{\theta ^{2} t_{1}^{2} }}{2}(xr + yq - bp)) \\ & - \frac{{C_{p} }}{{\theta ^{2} }}\left( {\left( {e^{{\theta t_{1} }} - 1} \right)\left( {a\theta - \left( {xr + yq - bp} \right)} \right)} \right. + t_{1} \theta \left( {xr + yq - bp} \right)e^{{\theta t_{1} }} \\ & \left. { + a\theta ^{2} \left( {T - t_{1} } \right) + \frac{{\theta ^{2} \left( {xr + yq - bp} \right)}}{2}\left( {T^{2} - t_{1}^{2} } \right)} \right) \\ & - C_{0} - \frac{{C_{d} }}{{\theta ^{2} }}\left( {\left( {e^{{\theta t_{1} }} - 1} \right)\left( {a\theta - \left( {xr + yq - bp} \right)} \right) - \theta t_{1} \left( {xr + yq - bp} \right)e^{{\theta t_{1} }} } \right. \\ & \left. { - \theta ^{2} \left( {at_{1} + \frac{{\left( {xr + yq - bp} \right)t_{1}^{2} }}{2}} \right)} \right) + C_{b} \left( {at_{1} \left( {T - t_{1} } \right) - \frac{a}{2}\left( {T^{2} - t_{1}^{2} } \right)} \right. \\ & \left. { + \frac{{\left( {xr + yq - bp} \right)}}{6}\left( {3t_{1}^{2} \left( {T - t_{1} } \right) - \left( {T^{3} - t_{1}^{3} } \right)} \right)} \right) \\ & + C_{p} I_{e} \left( {3at_{1} \left( {2D - t_{1} } \right) + \left( {xr + yq - bp} \right)t_{1}^{2} \left( {3D - t_{1} } \right)} \right) - AT\bigg] \\ \end{aligned}$$

The total profit of the inventory system assessed per unit of time throughout the planning cycle for both the payment delays channel in AM technology expenditure.

## Methodology

This section examines the optimal solution for sustainable inventory management, focusing on investments in additive manufacturing technology and credit options.

A function denoted as $\Gamma \left(t\right)=\frac{G\left(t\right)}{H\left(t\right)}, {t}_{1}\in {R}^{n}$ is strictly pseudo-concave under the condition that not only is the function $I(t)$ differentiable but also strictly concave. Additionally, $J(t)$ is infinite and strictly adheres to this characteristic. The outcome is derived from the analysis of the function $\Gamma \left(t\right)$.

### Theorem 1

*If* $$(e^{{\theta t_{1} }} (a\theta -$$$$(xr + yq - bp))$$$$\left( {\frac{{C_{h} }}{\theta } + C_{p} + C_{d} } \right)$$$$+ \left( {xr + yq - bp} \right)e^{{\theta t_{1} }}$$$$\left( {t_{1} \theta + 2} \right)$$$$\left( {\frac{{C_{h} }}{\theta } - C_{p} - C_{d} } \right)$$$$+ \frac{{C_{p} I_{p} }}{{\theta ^{3} }}((t_{1}$$$$- D)e^{{\left( {t_{1} - D} \right)}}$$$$(a\theta - (xr$$$$+ yq - bp)))$$$$+ a(C_{b} -$$$$C_{p} I_{p} - C_{p} I_{e} )$$$$+ \left( {xr + yq - bp} \right)$$$$((T - 2t_{1} ($$$$C_{p} I_{p} - C_{p} ))$$$$\left. {\left. { - C_{p} I_{e} e^{{\theta t_{1} }} } \right)} \right)$$$$\left( {a\left( {C_{b} - C_{p} I_{p} } \right)} \right.$$$$+ \left( {xr + yq - bp} \right)$$$$\left( {T\left( {C_{b} - C_{p} I_{p} } \right)} \right.$$$$\left. {\left. { + C_{p} - p} \right)} \right)$$$$> \left( {\left( {C_{b} - C_{p} I_{p} } \right)} \right.$$$$(a + t_{1} (xr+$$$$\left. {\left. {  yp - bp)} \right)} \right)^2$$ *The Hessian matrix pertaining to TP*_*1*_*(t*_*1*_*, T) demonstrates negative definiteness, thereby establishing that achieves a global maximum at the coordinates (t*_*1*_∗*, T*∗*). Importantly, the coordinates (t*_*1*_∗*, T*∗*) represent a singular and unique point of attainment.*

### Proof.:

Let us consider

$$\begin{aligned} G\left( {t_{1} ,T} \right) = & \frac{1}{T}\left[ {p\left( {aT + \frac{{\left( {xr + yq - bp} \right)}}{2}T^{2} } \right) - \frac{{C_{h} }}{{\theta ^{3} }}\left( {\left( {e^{{\theta t_{1} }} - 1} \right)(a\theta - (xr + yq - bp)} \right)} \right. \\ & - \theta t_{1} \left( {a\theta - \left( {xr + yq - bp} \right)e^{{\theta t_{1} }} } \right) - \frac{{\theta ^{2} t_{1}^{2} }}{2}(xr + yq - bp)) \\ & - \frac{{C_{p} }}{{\theta ^{2} }}\left( {\left( {e^{{\theta t_{1} }} - 1} \right)\left( {a\theta - \left( {xr + yq - bp} \right)} \right) + t_{1} \theta \left( {xr + yq - bp} \right)e^{{\theta t_{1} }} } \right. \\ & \left. { + a\theta ^{2} \left( {T - t_{1} } \right) + \frac{{\theta ^{2} \left( {xr + yq - bp} \right)}}{2}\left( {T^{2} - t_{1}^{2} } \right)} \right) \\ & - C_{0} - \frac{{C_{d} }}{{\theta ^{2} }}\left( {\left( {e^{{\theta t_{1} }} - 1} \right)\left( {a\theta - \left( {xr + yq - bp} \right)} \right)} \right. \\ & \left. { - \theta t_{1} \left( {xr + yq - bp} \right)e^{{\theta t_{1} }} - \theta ^{2} \left( {at_{1} + \frac{{\left( {xr + yq - bp} \right)t_{1}^{2} }}{2}} \right)} \right) \\ & + C_{b} \left( {at_{1} \left( {T - t_{1} } \right) - \frac{a}{2}\left( {T^{2} - t_{1}^{2} } \right) + \frac{{\left( {xr + yq - bp} \right)}}{6}\left( {3t_{1}^{2} \left( {T - t_{1} } \right) - \left( {T^{3} - t_{1}^{3} } \right)} \right)} \right. \\ & - \frac{{C_{p} I_{p} }}{{\theta ^{3} }}\left( {\left( {e^{{\left( {t_{1} - D} \right)}} - 1} \right)\left( {a\theta - \left( {xr + yq - bp} \right)} \right) + \theta \left( {xr + yq - bp} \right)t_{1} } \right. \\ & - \theta \left( {a\theta - \left( {xr + yq - bp} \right)} \right)\left( {t_{1} - D} \right) - \frac{{\theta ^{2} \left( {xr + yq - bp} \right)}}{2}\left( {t_{1}^{2} - D^{2} } \right) \\ & \left. { + \frac{{\theta ^{3} \left( {T - t_{1} } \right)}}{6}\left( {3a\left( {t_{1} - T} \right) + \left( {xr + yq - bp} \right)r\left( {2t_{1}^{2} - T^{2} - Tt_{1} } \right)} \right)} \right) \\ & + C_{p} I_{e} \left( {\frac{{at_{1}^{2} }}{2} + \left( {xr + yq - bp} \right)\frac{{t_{1}^{3} }}{3}} \right) - AT \Bigg]\\ \end{aligned}$$

and

20 $$H\left({t}_{1},T\right)=T$$

Partial differentiation with respect to both ${t}_{1}$ and $T$ of the Eq. (20)

21 $$\begin{aligned} \frac{{\partial G\left( {t_{1} ,T} \right)}}{{\partial t_{1} }} = & \left[ { - \frac{{C_{h} }}{{\theta ^{3} }}\left( {\theta e^{{\theta t_{1} }} \left( {a\theta - \left( {xr + yq - bp} \right)} \right) - \theta \left( {a\theta - \left( {xr + yq - bp} \right)\left( {t_{1} \theta e^{{\theta t_{1} }} + e^{{\theta t_{1} }} } \right)} \right) - \theta ^{2} \left( {xr + yq - bp} \right)t_{1} } \right)} \right. \\ & - \frac{{C_{p} }}{{\theta ^{2} }}\left( {\theta e^{{\theta t_{1} }} \left( {a\theta - \left( {xr + yq - bp} \right)} \right) + \theta \left( {xr + yq - bp} \right)\left( {t_{1} \theta e^{{t_{1} }} + e^{{\theta t_{1} }} } \right)} \right. \\ & - a\theta ^{2} - \theta ^{2} \left( {xr + yq - bp} \right)t_{1} - \frac{{C_{d} }}{{\theta ^{2} }}\left( {\theta e^{{\theta t_{1} }} \left( {a\theta - \left( {xr + yq - bp} \right)} \right)} \right. \\ & \left. { - \theta \left( {xr + yq - bp} \right)\left( {t_{1} \theta e^{{t_{1} }} + e^{{\theta t_{1} }} } \right) - \theta ^{2} a - \left( {xr + yq - bp} \right)t_{1} } \right) \\ & \left. { + C_{b} \left( {aT - at_{1} - \frac{{xr + yq - bp}}{6}} \right)\left( {6Tt_{1} - 6t_{1}^{2} } \right)} \right) \\ & - \frac{{C_{p} I_{p} }}{{\theta ^{3} }}\left( {\left( {t_{1} - D} \right)e^{{\left( {t_{1} - D} \right)}} \left( {a\theta - \left( {xr + yq - bp} \right)} \right)} \right. \\ & + \theta \left( {xr + yq - bp} \right) + \theta \left( {a\theta - \left( {xr + yq - bp} \right)} \right) \\ & - \theta ^{2} \left( {xr + yq - bp} \right) + \frac{{\theta ^{3} }}{6}  {\left( {\left( {6aT - 6at_{1} } \right) + \left( {xr + yq - bp} \right)\left( {6Tt_{1} - 6t_{1}^{2} } \right)} \right)}  \\ & + C_{p} I_{e} \left( {at_{1} + t_{1}^{2} \left( {xr + yq - bp} \right)} \right)\bigg] \\ \end{aligned}$$

Again, partial differentiation concerning both ${t}_{1}$ and $T$ of Eq. (21), we can derive

22 $$\begin{aligned} \frac{{\partial ^{2} G\left( {t_{1} ,T} \right)}}{{\partial t_{1}^{2} }} = &  \left[{ - e^{{\theta t_{1} }} \left( {a\theta - \left( {xr + yq - bp} \right)} \right)\left( {\frac{{C_{h} }}{\theta } + C_{p} + C_{d} } \right)} \right. \\ & - \left( {xr + yq - bp} \right)e^{{\theta t_{1} }} \left( {t_{1} \theta + 2} \right)\left( {\frac{{C_{h} }}{\theta } + C_{p} - C_{d} } \right) - \left( {xr + yq - bp} \right)\left( {\frac{{C_{h} }}{\theta } - C_{p} - C_{d} } \right) \\ & - \frac{{C_{p} I_{p} }}{{\theta ^{3} }}\left( {\left( {t_{1} - D} \right)e^{{t_{1} - D}} \left( {a\theta - \left( {xr + yq - bp} \right)} \right)} \right) - a\left( {C_{b} - C_{p} I_{p} - C_{p} I_{e} } \right) \\ &  { - \left( {xr + yq - bp} \right)\left( {\left( {T - 2t_{1} } \right)\left( {C_{p} I_{p} - C_{b} } \right) - C_{p} I_{e} 2t_{1} } \right)}  \bigg] \\ \end{aligned}$$

23 $$\text{and }\frac{{\partial }^{2}G\left({t}_{1},T\right)}{\partial T\partial {t}_{1}}=\left({C}_{b}-{C}_{p}{I}_{p}\right)\left(a+{t}_{1}\left(xr+yp-bp\right)\right)$$

By taking the partial derivative of Eq. (19) with respect to $T$, we can deduce

24 $$\begin{aligned} \frac{{\partial G\left( {t_{1} ,T} \right)}}{{\partial T}} = & p\left( {a + \left( {xr + yq - bp} \right)T}) \right) - C_{p} \left( {a + \left( {xr + yq - bp} \right)T} ) \right. \\ & + C_{b} \left( {at_{1} - aT} \right) + \frac{{\left( {xr + yq - bp} \right)}}{6}\left( {3t_{1}^{2} - 3T^{2} } \right) \\ &  { - \frac{{C_{p} I_{p} }}{6}\left( {6at_{1} - 6aT + \left( {xr + yq - bp} \right)\left( {3t_{1}^{2} - 3T^{2} } \right)} \right)}  \\ \end{aligned}$$

Taking the partial derivative of Eq. (24) with respect to T allows for the derivation of

25 $$\frac{{\partial }^{2}G\left({t}_{1},T\right)}{\partial {T}^{2}}=-a\left({C}_{b}-{C}_{p}{I}_{p}\right)-\left(xr+yq-bp\right)\left(\left({C}_{p}-p\right)+T\left({C}_{b}-{C}_{p}{I}_{p}\right)\right)$$

26 $$\frac{{\partial }^{2}G\left({t}_{1},T\right)}{\partial {t}_{1}\partial T}=\left({C}_{b}-{C}_{p}{I}_{p}\right)\left(a+{t}_{1}\left(xr+yp-bp\right)\right)$$

The Hessian matrix for $G\left({t}_{1},T\right)$ is given by $$\left[\begin{array}{cc}\frac{\partial G\left({t}_{1},T\right)}{\partial {t}_{1}^{2}}& \frac{\partial G\left({t}_{1},T\right)}{\partial {t}_{1}\partial T}\\ \frac{\partial G\left({t}_{1},T\right)}{\partial T\partial {t}_{1}}& \frac{\partial G\left({t}_{1},T\right)}{\partial {T}^{2}}\end{array}\right]$$ The first principal minor is:

27 $$\frac{{\partial }^{2}G\left({t}_{1},T\right)}{\partial {T}^{2}}=-a\left({C}_{b}-{C}_{p}{I}_{p}\right)-\left(xr+yq-bp\right)\left(\left({C}_{p}-p\right)+T\left({C}_{b}-{C}_{p}{I}_{p}\right)\right)<0$$

Even more, the second principal minor is:

$$\begin{aligned} \left[ {\begin{array}{*{20}c} {\frac{{\partial G\left( {t_{1} ,T} \right)}}{{\partial t_{1}^{2} }}} & {\frac{{\partial G\left( {t_{1} ,T} \right)}}{{\partial t_{1} \partial T}}} \\ {\frac{{\partial G\left( {t_{1} ,T} \right)}}{{\partial T\partial t_{1} }}} & {\frac{{\partial G\left( {t_{1} ,T} \right)}}{{\partial T^{2} }}} \\ \end{array} } \right] = & \frac{{\partial ^{2} G\left( {t_{1} ,T} \right)}}{{\partial t_{1}^{2} }}\frac{{\partial ^{2} G\left( {t_{1} ,T} \right)}}{{\partial T^{2} }} - \frac{{\partial ^{2} G\left( {t_{1} ,T} \right)}}{{\partial t_{1} \partial T}}\frac{{\partial ^{2} G\left( {t_{1} ,T} \right)}}{{\partial T\partial t_{1} }} \\ & =  {e^{{\theta t_{1} }} \left( {a\theta - \left( {xr + yq - bp} \right)} \right)\left( {\frac{{C_{h} }}{\theta } + C_{p} + C_{d} } \right) + \left( {xr + yq - bp} \right)}  \\ & e^{{\theta t_{1} }} \left( {t_{1} \theta + 2} \right)\left( {\frac{{C_{h} }}{\theta } - C_{p} - C_{d} } \right) + \frac{{C_{p} I_{p} }}{{\theta ^{3} }}\left( {\left( {t_{1} - D} \right)e^{{\left( {t_{1} - D} \right)}} \left( {a\theta - \left( {xr + yq - bp} \right)} \right)} \right) \\ & \left. { + a\left( {C_{b} - C_{p} I_{p} - C_{p} I_{e} } \right) + \left( {xr + yq - bp} \right)\left( {\left( {T - 2t_{1} } \right)\left( {C_{p} I_{p} - C_{p} } \right) - C_{p} I_{e} e^{{\theta t_{1} }} } \right)} \right) \\ & \left( {a\left( {C_{b} - C_{p} I_{p} } \right) + \left( {xr + yq - bp} \right)\left( {T\left( {C_{b} - C_{p} I_{p} } \right) + C_{p} - p} \right)} \right) \\ & - \left( ({C_{b} - C_{p} I_{p} } \right)\left( {a + t_{1} \left( {xr + yp - bp} \right)} )\right)^{2}  \\ \end{aligned}$$

The second principal minor is > 0 only if $$(e^{{\theta t_{1} }} \left( {a\theta - \left( {xr + yq - bp} \right)} \right)$$$$\left( {\frac{{C_{h} }}{\theta } + C_{p} + C_{d} } \right)$$$$+ \left( {xr + yq - bp} \right)$$$$e^{{\theta t_{1} }} \left( {t_{1} \theta + 2} \right)$$$$\left( {\frac{{C_{h} }}{\theta } + C_{p} - C_{d} } \right)$$$$+ \frac{{C_{p} I_{p} }}{{\theta ^{3} }}(\left( {t_{1} - D} \right)$$$$e^{{t_{1} - D}} (a\theta$$$$- \left( {xr + yq - bp} \right)))$$$$+ a(C_{b} - C_{p} I_{p} - C_{p} I_{e} )$$$$+ \left( {xr + yq - bp} \right)$$$$\left( {\left( {T - 2t_{1} } \right)} \right.\left( {C_{p} I_{p} - C_{b} } \right)$$$$\left. {\left. { - C_{p} I_{e} e^{{\theta t_{1} }} } \right)} \right)$$$$(a\left( {C_{b} - C_{p} I_{p} } \right)$$$$+ \left( {xr + yq - bp} \right)$$$$(T(C_{b} - C_{p} I_{p} )$$$$+ C_{p} - p))$$$$> \left(( {C_{b} - C_{p} I_{p} } \right)$$$$(a + t_{1}$$$$\left( {xr + yp - bp}) )\right)^{2}$$

Given that $\frac{{\partial }^{2} \mathrm{G}\left({\mathrm{t}}_{1},\mathrm{T}\right)}{\partial {\mathrm{t}}_{1}^{2}}<0$ and ${H}_{\mathrm{2,2}}>0$, the function $G\left({t}_{1},T\right)$ exhibits strict concavity and differentiability. Moreover, $G\left({t}_{1},T\right)$ represents a strictly positive affine function. According to theorem 3.2.5 from^55^, $T{P}_{1}\left({t}_{1},T\right)$ attains the global maximum value at a specific point under the specified conditions, denoted as $\left({t}_{1}^{*},{T}^{*}\right).$ This concludes the proof.

**Figure:**
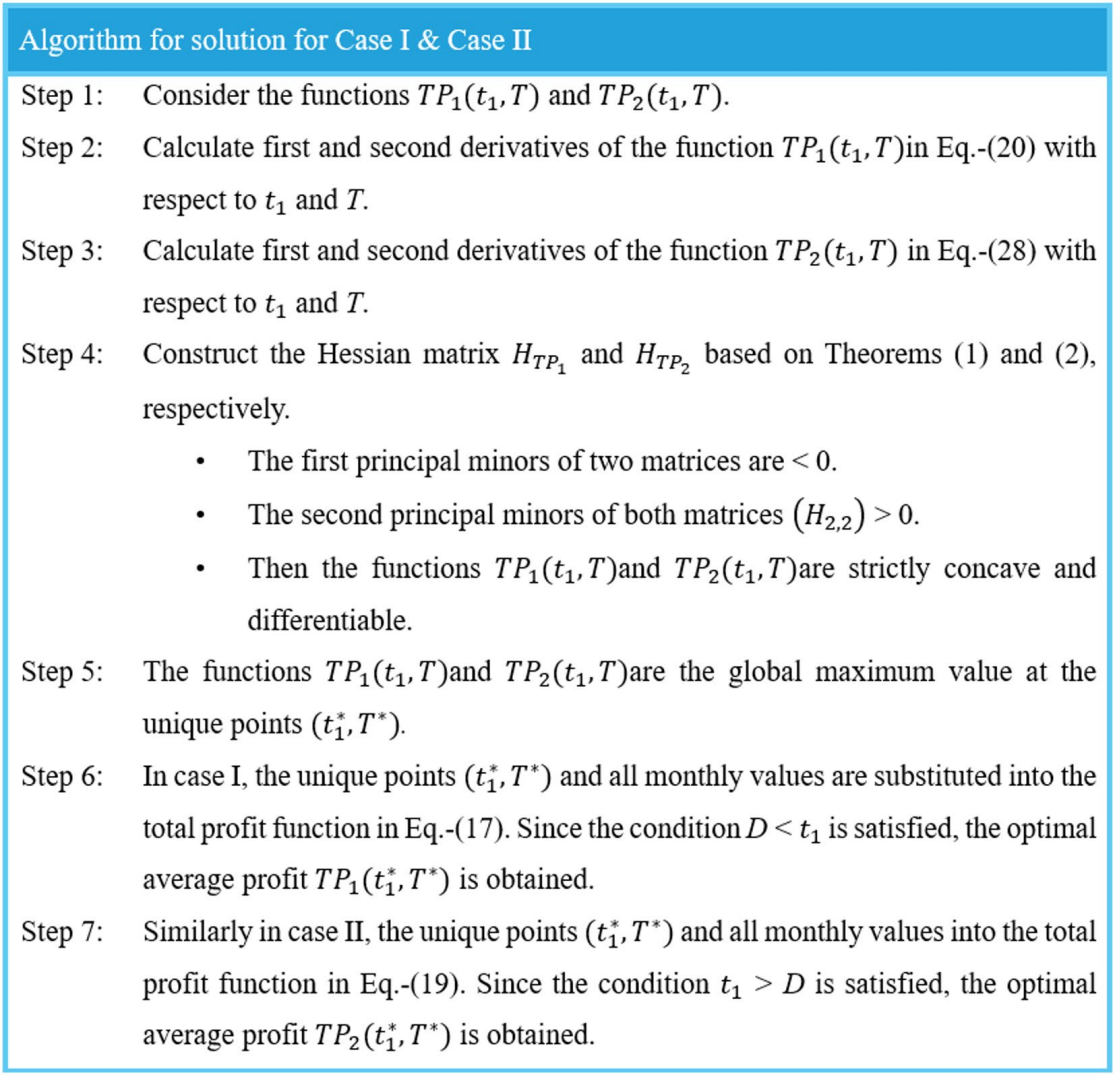
Algorithm for solution for Case I & Case II

### Theorem 2

*If* $$(e^{{\theta t_{1} }} (a\theta -$$$$(xr + yq - bp))$$$$\left( {\frac{{C_{h} }}{\theta } + C_{p} + C_{d} } \right)$$$$+ e^{{\theta t_{1} }} \left( {t_{1} \theta + 2} \right)$$$$\left( {xr + yq - bp} \right)$$$$\left( {\frac{{C_{h} }}{\theta } + C_{p} - C_{d} } \right)$$$$+ \left( {xr + yq - bp} \right)$$$$\left( { - \frac{{C_{h} }}{\theta } + C_{p} - C_{d} } \right)$$$$+ a\left( {C_{b} + 6C_{p} I_{e} } \right)$$$$+ \left( {xr + yq - bp} \right)$$$$(C_{b} \left( {T - 2t_{1} } \right)$$$$+ 6C_{p} I_{e} (D - t_{1} ))$$$$(C_{b} a + \left( {xr + yq - bp} \right)$$$$\left( {p - C_{p} - C_{b} T} \right)))$$$$> (C_{b} (a + t_{1}$$$$(xr + yp - bp)))^{2}$$. *The Hessian matrix associated with* $$T{P}_{2}\left({t}_{1},T\right)$$ *consistently exhibits negative definiteness, signifying that it attains its global maximum at the singular point *$$\left({t}_{1}^{*},{T}^{*}\right)$$*, and this extremum is unique.*

### Proof:

Let us consider

28 $$\begin{aligned} G\left( {t_{1} ,T} \right) = & \frac{1}{{\mathrm{T}}}\left[ {p\left( {aT + \frac{{\left( {xr + yq - bp} \right)}}{2}T^{2} } \right) - \frac{{C_{h} }}{{\theta ^{3} }}\left( {\left( {e^{{\theta t_{1} }} - 1} \right)(a\theta - (xr + yq - bp)} \right)} \right. \\ & - \theta t_{1} \left( {a\theta - \left( {xr + yq - bp} \right)e^{{\theta t_{1} }} } \right) - \frac{{\theta ^{2} t_{1}^{2} }}{2}(xr + yq - bp)) \\ & - \frac{{C_{p} }}{{\theta ^{2} }}\left( {\left( {e^{{\theta t_{1} }} - 1} \right)\left( {a\theta - \left( {xr + yq - bp} \right)} \right)} \right. \\ & + t_{1} \theta \left( {xr + yq - bp} \right)e^{{\theta t_{1} }} + a\theta ^{2} \left( {T - t_{1} } \right) \\ & \left. { + \frac{{\theta ^{2} \left( {xr + yq - bp} \right)}}{2}\left( {T^{2} - t_{1}^{2} } \right)} \right) \\ & - C_{0} - \frac{{C_{d} }}{{\theta ^{2} }}\left( {\left( {e^{{\theta t_{1} }} - 1} \right)\left( {a\theta - \left( {xr + yq - bp} \right)} \right) - \theta t_{1} \left( {xr + yq - bp} \right)e^{{\theta t_{1} }} } \right. \\ & \left. { - \theta ^{2} \left( {at_{1} + \frac{{\left( {xr + yq - bp} \right)t_{1}^{2} }}{2}} \right)} \right) \\ & + C_{b} \left( {at_{1} \left( {T - t_{1} } \right) - \frac{a}{2}\left( {T^{2} - t_{1}^{2} } \right) + \frac{{\left( {xr + yq - bp} \right)}}{6}\left( {3t_{1}^{2} \left( {T - t_{1} } \right) - \left( {T^{3} - t_{1}^{3} } \right)} \right)} \right) \\ & + C_{p} I_{e} \left( {3at_{1} \left( {2D - t_{1} } \right) + \left( {xr + yq - bp} \right)t_{1}^{2} \left( {3D - t_{1} } \right)} \right) - AT\bigg] \\ \end{aligned}$$

and

$$H\left({t}_{1},T\right)=T$$

Using partial differentiation with regard to ${t}_{1}$ and $T$ of Eq. (28), one can derive

29 $$\begin{aligned} \frac{{\partial G\left( {t_{1} ,T} \right)}}{{\partial t_{1} }} = & \left[ { - \frac{{C_{h} }}{{\theta ^{3} }}\left( {\theta e^{{\theta t_{1} }} \left( {a\theta - \left( {xr + yq - bp} \right)} \right) - \theta \left( {a\theta - \left( {xr + yq - bp} \right)\left( {t_{1} \theta e^{{\theta t_{1} }} + e^{{\theta t_{1} }} } \right)} \right) - \theta ^{2} \left( {xr + yq - bp} \right)t_{1} } \right)} \right. \\ & - \frac{{C_{p} }}{{\theta ^{2} }}\left( {\theta e^{{\theta t_{1} }} \left( {a\theta - \left( {xr + yq - bp} \right)} \right) + \theta \left( {xr + yq - bp} \right)\left( {t_{1} \theta e^{{t_{1} }} + e^{{\theta t_{1} }} } \right)} \right. \\ & - a\theta ^{2} - \theta ^{2} \left( {xr + yq - bp} \right)t_{1} - \frac{{C_{d} }}{{\theta ^{2} }}\left( { e^{{\theta t_{1} }} \left( {a\theta - \left( {xr + yq - bp} \right)} \right)} \right. \\ & \left. { - \theta \left( {xr + yq - bp} \right)\left( {t_{1} \theta e^{{t_{1} }} + e^{{\theta t_{1} }} } \right) - \theta ^{2} (a + \left( {xr + yq - bp} \right)t_{1} } \right) \\ & \left. { + C_{b} \left( {aT - at_{1} -{(xr + yq - bp}} \right)\left( {Tt_{1} - t_{1}^{2} } \right)} \right) \\ &    + C_{p} I_{e} \left( {6Da -6a t_{1}+ ( {xr + yq - bp}) \left( {6Dt_{1} - 6t_{1}^{2} } \right)} \right)\bigg] \\ \end{aligned}$$

Using partial differentiation with regard to both ${t}_{1}$ and $T$ of Eq. (29), we can find

30 $$\begin{aligned} \frac{{\partial ^{2} G\left( {t_{1} ,T} \right)}}{{\partial t_{1}^{2} }} = & \left[ { - e^{{\theta t_{1} }} \left( {a\theta - \left( {xr + yq - bp} \right)} \right)\left( {\frac{{C_{h} }}{\theta } + C_{p} + C_{d} } \right)} \right. \\ & - e^{{\theta t_{1} }} \left( {t_{1} \theta + 2} \right)\left( {xr + yq - bp} \right)\left( {\frac{{C_{h} }}{\theta } + C_{p} - C_{d} } \right)r \\ & - \left( {xr + yq - bp} \right)\left( {\frac{{ - C_{h} }}{\theta } - C_{p} - C_{d} } \right) \\ & - a\left( {C_{b} + C_{p} - 6C_{p} I_{e} } \right) - \left( {xr + yq - bp} \right) \\ &  {\left( {C_{b} \left( {T - 2t_{1} } \right) - 6C_{p} I_{e} \left( {6D - 6t_{1} } \right)} \right)}  \bigg] \\ \end{aligned}$$

31 $$\text{and }\frac{{\partial }^{2}G\left({t}_{1},T\right)}{\partial T\partial {t}_{1}}={C}_{b}\left(a+\left(xr+yp-bp\right){t}_{1}\right)$$

By partially differentiating Eq. (28) with regard to T, we can obtain

32 $$\frac{\partial G\left({t}_{1},T\right)}{\partial T}=p\left(a+\left(xr+yq-bp\right)T\right)+{C}_{b}\left(a{t}_{1}-aT+\frac{\left(xr+yq-bp\right)}{6}\left(3{t}_{1}^{2}-3{T}^{2}\right)\right)-{C}_{p}\left(xr+yq-bp\right)$$

Taking the partial derivative of Eq. (32) with respect to T, can derive

33 $$\frac{{\partial }^{2}G\left({t}_{1},T\right)}{\partial {T}^{2}}=-\left({C}_{b}a+\left(xr+yq-bp\right)\left(p-{C}_{p}-{C}_{b}T\right)\right)$$

The Hessian matrix for $G\left({t}_{1},T\right)$ is given by $$\left[\begin{array}{cc}\frac{\partial G\left({t}_{1},T\right)}{\partial {t}_{1}^{2}}& \frac{\partial G\left({t}_{1},T\right)}{\partial {t}_{1}\partial T}\\ \frac{\partial G\left({t}_{1},T\right)}{\partial T\partial {t}_{1}}& \frac{\partial G\left({t}_{1},T\right)}{\partial {T}^{2}}\end{array}\right]$$ The first principal minor is:

$$\left[\begin{array}{cc}\frac{\partial G\left({t}_{1},T\right)}{\partial {t}_{1}^{2}}& \frac{\partial G\left({t}_{1},T\right)}{\partial {t}_{1}\partial T}\\ \frac{\partial G\left({t}_{1},T\right)}{\partial T\partial {t}_{1}}& \frac{\partial G\left({t}_{1},T\right)}{\partial {T}^{2}}\end{array}\right]=\frac{\partial { }^{2}G\left({t}_{1},T\right)}{\partial {t}_{1}^{2}} \frac{{\partial }^{2}G\left({t}_{1},T\right)}{\partial {T}^{2}}- \frac{{\partial }^{2}G\left({t}_{1},T\right)}{\partial {t}_{1}\partial T}\frac{{\partial }^{2}G\left({t}_{1},T\right)}{\partial T\partial {t}_{1}}$$

The second principal minor is positive > 0 only if

$$\begin{aligned} & \left( {e^{{\theta t_{1} }} \left( {a\theta - \left( {xr + yq - bp} \right)} \right)\left( {\frac{{C_{h} }}{\theta } + C_{p} + C_{d} } \right)} \right. \\ & \quad + \left( {xr + yq - bp} \right)\left( { - \frac{{C_{h} }}{\theta } + C_{p} - C_{d} } \right) \\ & \quad + a\left( {C_{b} + 6C_{p} I_{e} } \right) + \left( {xr + yq - bp} \right) \\ & \quad \left( {C_{b} \left( {T - 2t_{1} } \right) + 6C_{p} I_{e} \left( {D - t_{1} } \right)} \right) \\ & \quad  {\left( {C_{b} a + \left( {xr + yq - bp} \right)\left( {p - C_{p} - C_{b} T} \right)} \right)}  \bigg) \\ & \quad - \left( {C_{b} \left( {a + t_{1} \left( {xr + yp - bp} \right)} \right)}  \right)^{2} > 0 \\ \end{aligned}$$

Since $\frac{{\partial }^{2}T{P}_{2}\left({t}_{1},T\right)}{\partial {t}_{1}^{2}}<0$ and ${H}_{\mathrm{2,2}}>0$, the function $G\left({t}_{1},T\right)$ is both strictly concave and differentiable.

In addition, the function *H* $\left({t}_{1},T\right)=T$ to is strictly positive and affine. Based on theorem 3.2.5 of^55^, $T{P}_{2}\left({t}_{1},T\right)$ attains its global maximum at the unique point determined by the necessary conditions, specifically, it is $\left({t}_{1}^{*},{T}^{*}\right)$. This concludes the proof.

## Numerical illustration

**Fig. 2:**
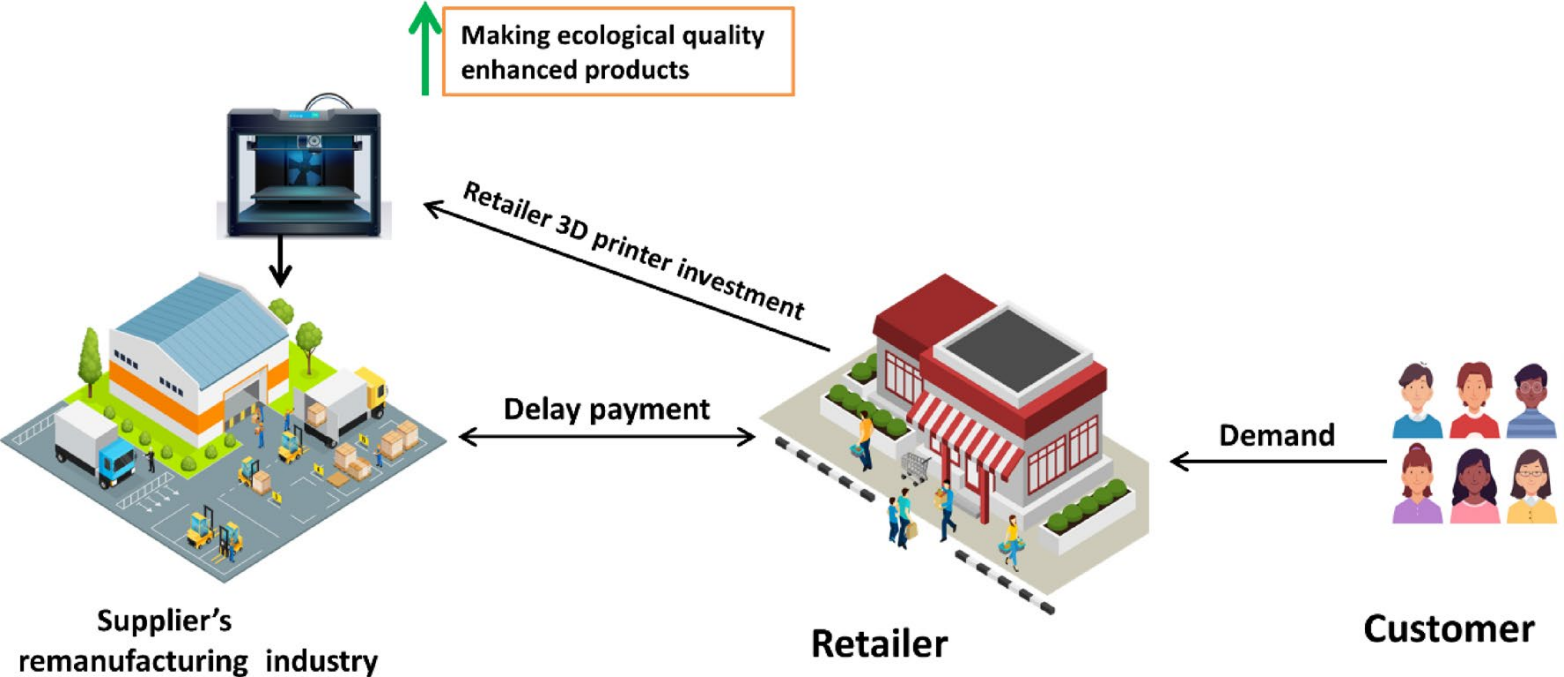
Illustration of interlink and strategy of proposed inventory system.

**Fig. 3:**
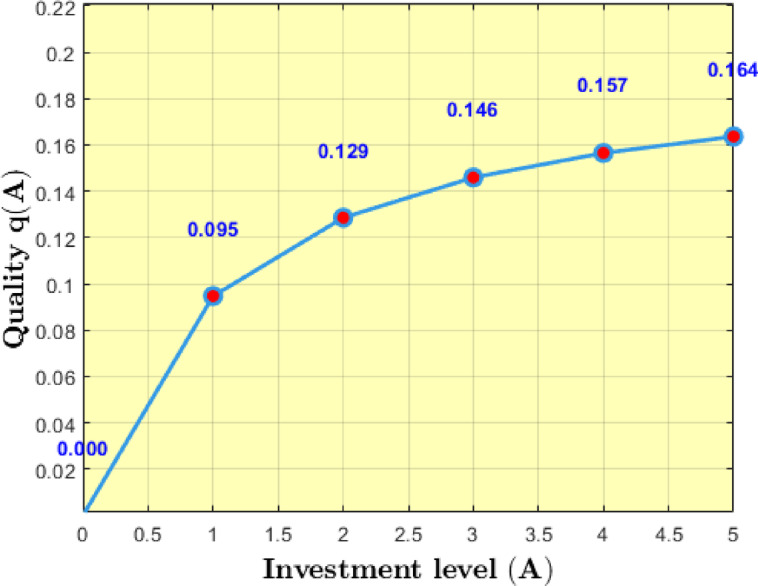
Exhibit dependence of product quality on the investment level.

This section presents a case study of a retailer implementing 3D printing technology to manufacture products from recyclable plastic, with a focus on enhancing product quality and reliability and environmental considerations. To assess the effectiveness of the proposed inventory model, realistic operational data reflecting typical industry conditions are used, demonstrating how the model supports informed decision-making and improves overall operational performance. This approach highlights the model’s practical relevance and applicability. The conceptual framework, illustrated in Fig. 2, depicts the retailer’s inventory system, emphasizing investment in 3D printing to transform recyclable plastic into high-quality products while simultaneously increasing profitability (Fig. 3).

Consider the various types of cost components that retailers can use to develop a sustainable inventory system. Examine the different cost components incurred by the retailer in establishing a sustainable inventory management system. The retailer spends for cost per purchasing unit item $${C}_{p}$$ is $20, ordering cost per order $${C}_{o}$$ is $500, cost of selling unit item is $$p$$ is $70, holding cost per unit time per unit item $${C}_{h}$$ is $1.5, shortage cost per unit time per unit item $${C}_{b}$$ is $12 and deterioration cost per decaying goods per unit per unit time $${C}_{d}$$ is $8. The initial deterioration rate of the system *θ* is 0.2. The demand function $$R(p,r,q)$$ having initial demand *a* is 100, $$x = 30, y = 20$$, the coefficient of price sensitivity $$b$$ is 0.5, AM investment for quality products A is $5, efficiency of the AM technology $$e$$ is 0.9, amount of recycling products $$u$$ is 0.9, supplier’s quality of the products s is 0.9 and $${a}_{0}= 2.5$$.

In practical supply chain systems, suppliers commonly provide trade credit to retailers to encourage purchasing and maintain long-term commercial relationships. The credit period D represents the duration during which the retailer can postpone payment for purchased goods without incurring penalties. The length of this period is generally influenced by industry payment standards, supplier-retailer bargaining power, and inventory turnover cycles. In many retail sectors dealing with recycled polymer and consumer products, credit periods typically vary from a few weeks to several months. The proposed system also includes a delayed payment policy, where the retailer earns interest at a rate of 5% (*I*_*e*_), and the supplier incurs an interest cost of 7% (*I*_*p*_). The inventory system is analyzed over a one-year planning horizon, and by substituting the relevant parameter values into (16) and (18), the optimal value and total profit are derived. Table 2 summarizes the optimal average profit obtained for different lengths of the payment delay under both scenarios. In this analysis, the supplier is assumed to provide multiple credit-period alternatives ranging from two months up to one year, namely (i) two months, (ii) four months, (iii) six months, (iv) nine months, and (v) twelve months.

**Table 2:** Optimum total profit associated with varying delay payment.

Delay of payments	*t*_1_^*^ (year)	*T* = *T*_1_^*^	*TP*_1_^*^	*t*_1_^*^ (year)	*T* = *T*_2_^*^	*TP*_2_^*^
2 months (D = 0.1667)	0.5806	0.9951	3991	0.4683	0.9167	3943
3 months (D = 0.25)	0.5883	0.9972	3995	0.4859	0.9134	3969
4 months (D = 0.3333)	0.5965	1.0002	3999	0.5031	0.9094	3995
5 months (D = 0.4167)	0.6050	1.0040	4001	0.5198	0.9043	4022
6 months (D = 0.5)	0.6138	1.0086	4003	0.5360	0.8982	4051
7 months (D = 0.5833)	0.6229	1.0138	4004	0.5518	0.8913	4080
8 months (D = 0.6667)	0.6324	1.0199	4004	0.5672	0.8834	4110
9 months (D = 0.75)	0.6422	1.0265	4003	0.5821	0.8745	4142
10 months (D = 0.8333)	0.6522	1.0339	4001	0.5964	0.8646	4175
11 months (D = 0.9167)	0.6625	1.0418	3999	0.6105	0.8537	4208
1 year	0.6731	1.0504	3996	0.6239	0.8418	4243

**Fig. 4:**
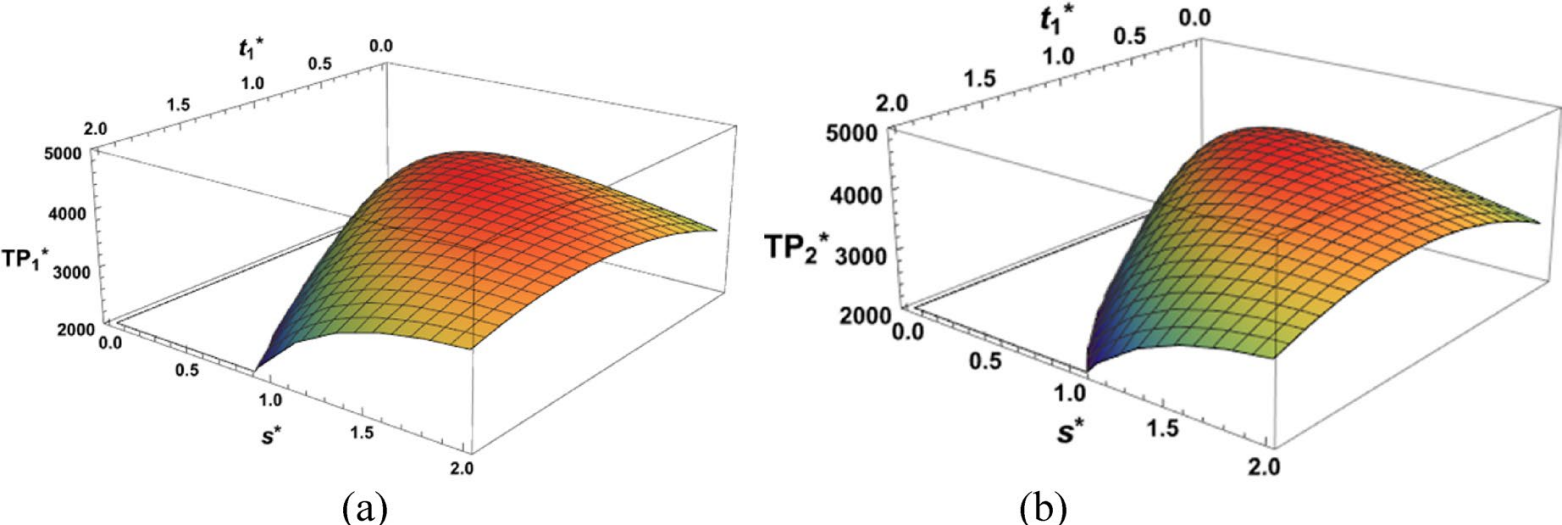
Graphical representation of the concavity of the optimal total profit with respect to ${t}_{1}^{*}$ and ${p}^{*}$. (**a**) and (**b**) shows the optimal total profit with and without investments in AM and green technologies by $T{P}_{1}^{*}$ and $T{P}_{2}^{*}$, respectively.

Table 2 presents the optimal decision variables and the corresponding total profits for different delayed payment periods. The results show how variations in the credit period D influence the retailer’s inventory cycle and profitability under both scenarios. When the credit period is relatively short, such as two months (D = 0.1667) and three months (D = 0.25), the condition $D<{t}_{1}$ holds, which corresponds to Case-I. Under this situation, the retailer must finance the remaining inventory after the credit period expires, and the maximum average profit is achieved under ${TP}_{1}$. As the credit period increases to four, five, and six months (D = 0.3333, 0.4167 and 0.5), $D<{t}_{1}$ still holds, and Case-I continues to govern the system. Consequently, the retailer’s profit under ${TP}_{1}$ gradually increases due to improved financial flexibility. When the credit period reaches seven months (D = 0.5833), both cases become feasible because the credit period lies between the two critical times. Therefore, the system provides two possible profit outcomes, ${{TP}_{1}}^{*}$ and ${{TP}_{2}}^{*}$. The highlighted row in Table 2 represents the optimal solution for the system. For longer credit periods, such as eight months to one year (D = 0.6667 to D = 1), the credit period becomes sufficiently large relative to the inventory cycle, and the model follows Case-II. Under this condition, the retailer can sell most or all of the inventory before the payment becomes due, which eliminates financing costs and allows the retailer to benefit from the delayed payment period. As a result, the total profit under ${TP}_{2}$ increases significantly as the credit period extends. However, the increase in profit gradually stabilizes because of the balancing effects of holding costs, deterioration, and demand within the EOQ framework. The concave representation of the optimal total profit under AM technology investment is shown in Fig. 4 for ${{TP}_{1}}^{*}$ and ${{TP}_{2}}^{*}$.

Table 3 presents a comparative analysis of the retailer’s optimal results with and without investment in AM technology. It can be observed that when the retailer incorporates AM particularly 3D printing to enhance product quality and reliability, the total profit increases notably. This improvement arises because AM enables the production of parts with higher precision, reduced defects, and better material utilization, leading to greater customer satisfaction and repeat demand. Although the production cycle time slightly decreases due to improved process efficiency, the profitability remains consistently higher compared with the conventional recycling approach.

**Table 3:** Assessment of Additive manufacturing on optimal solution.

Additive manufacturing	*t*_1_^*^ (year)	T^*^	Q^*^ = *Q*_1_	*TP*_1_^*^	*t*_1_^*^ (year)	T^*^	Q^*^ = *Q*_1_	*TP*_2_^*^
With	0.6229	1.0138	98.9513	4004	0.5518	0.8913	96.3456	4080
Without	0.5680	0.9154	87.3020	3896	0.5518	0.8160	85.2948	3983

## Sensitivity analysis

The purpose of this investigation is to examine the behaviour of parameters and to evaluate the applicability of theoretical findings in the context of recycling plastic products in companies or industries. Implementing a 3D technology-based inventory model has as its primary objective to maximize profits through cost reduction, as well as the improvement of product quality and reliability. The analysis will consider the observed variations in these parameter values ${C}_{0},{C}_{h},{C}_{p},{C}_{b},{C}_{d}$.

**Fig. 5:**
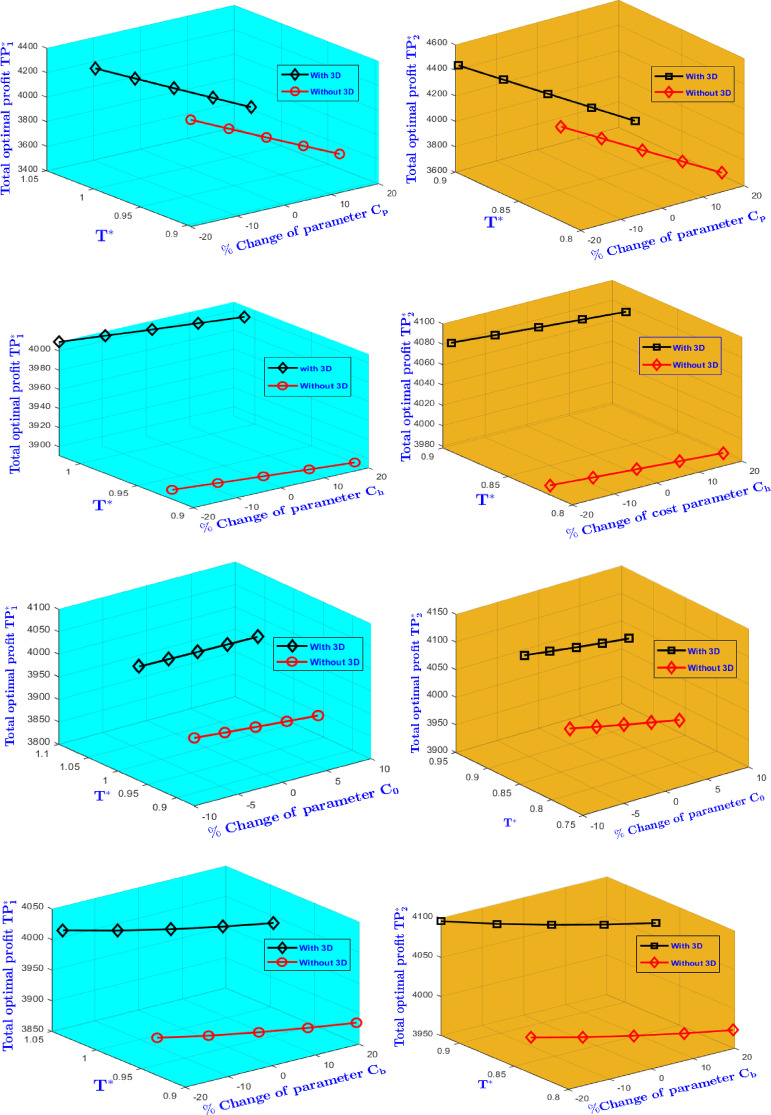
Impact of cost parameters on total optimal profit incorporating delay payment scenario for with and without AM technology.

From Table 4, it is noted that the ordering cost ${C}_{O}$ of the sensitivity is high. If ordering cost, the times ${t}_{1}^{*}$ and ${T}_{1}^{*}$ are increases and the total profits ${TP}_{1}^{*}$ and ${TP}_{2}^{*}$ are decreases, mean while the time ${t}_{2}^{*}$ and ${T}_{2}^{*}$ are increasing the total profits ${TP}_{1}^{*}$ and ${TP}_{2}^{*}$ are decreasing. The holding cost ${C}_{h}$ is less responsive to overall profit than cycle times. Increases in the holding cost result in a simultaneous decrease in both overall profit and cycle duration, a prevalent phenomenon frequently observed in the domain of inventory management (Fig. 5).

**Table 4:** Impact of cost parameters on variables and total costs via sensitivity analysis by evaluating % of change.

Cost	%change	*t*_1_^*^ (year)	%*t*_1_*	*T*_1_^*^ (year)	%*T*_1_*	*TP*_1_^*^	%*TP*_1_^*^	*t*_2_^*^ (year)	%*t*_2_*	*T*_2_^*^ (year)	%*T*_2_*	*TP*_2_^*^	%*TP*_2_^*^
${C}_{O}$	− 12	0.5808	− 9.34	0.9525	− 10.29	4065	2.60	0.5248	− 8.35	0.8319	− 11.37	4150	2.92
	− 10	0.5946	− 4.54	0.9630	− 5.01	4054	1.25	0.5295	− 4.04	0.8421	− 5.52	4138	1.42
	10	0.6500	4.35	1.0624	4.79	3956	− 1.20	0.5731	3.86	0.9381	5.25	4025	− 1.35
	12	0.6758	8.49	1.1089	9.38	3909	− 2.37	0.5934	7.54	0.9828	10.27	3973	− 2.62
${C}_{p}$	− 20	0.6422	3.10	0.9979	− 1.57	4389	9.62	0.5735	3.93	0.8972	0.66	4450	9.07
	− 10	0.6323	1.51	1.0055	− 0.82	4196	4.80	0.5621	1.87	0.8940	0.30	4265	4.53
	10	0.6142	− 1.40	1.0228	0.89	3811	− 4.82	0.5426	− 1.67	0.8890	− 0.26	3895	− 4.53
	20	0.6060	− 2.71	1.0324	1.83	3620	− 9.59	0.5343	− 3.17	0.8872	− 0.46	3710	− 9.07
${C}_{h}$	− 20	0.6363	2.15	1.0185	0.46	4009	0.12	0.5595	1.40	0.8933	0.22	4085	0.12
	− 10	0.6295	1.06	1.0161	0.23	4007	0.07	0.5556	0.69	0.8923	0.11	4082	0.05
	10	0.6165	− 1.03	1.0116	− 0.22	4001	− 0.07	0.5481	− 0.67	0.8903	− 0.11	4077	− 0.07
	20	0.6102	− 2.04	1.0094	− 0.43	3998	− 0.15	0.5444	− 1.34	0.8893	− 0.22	4075	− 0.12
${C}_{b}$	− 20	0.5781	− 7.91	1.0374	2.33	4023	0.47	0.5279	− 4.33	0.9163	2.80	4096	0.39
	− 10	0.6024	− 3.29	1.0246	1.07	4013	0.22	0.5407	− 2.01	0.9029	1.30	4087	0.17
	10	0.6407	2.86	1.0046	− 0.91	3996	− 0.20	0.5617	1.79	0.8810	− 1.16	4073	− 0.17
	20	0.6561	5.33	0.9965	− 1.71	3989	− 0.37	0.5705	3.39	0.8721	− 2.15	4067	− 0.32
${C}_{d}$	− 20	0.6371	2.28	1.0188	0.49	4010	0.15	0.5600	1.49	0.8935	0.25	4085	0.12
	− 10	0.6300	1.14	1.0163	0.25	4007	0.07	0.5559	0.74	0.8923	0.11	4083	0.07
	10	0.6161	− 1.09	1.0114	− 0.24	4001	− 0.07	0.5479	− 0.71	0.8902	− 0.12	4077	− 0.07
	20	0.6094	− 2.17	1.0091	− 0.46	3998	− 0.15	0.5439	− 1.43	0.8892	− 0.24	4075	− 0.12

**Fig. 6:**
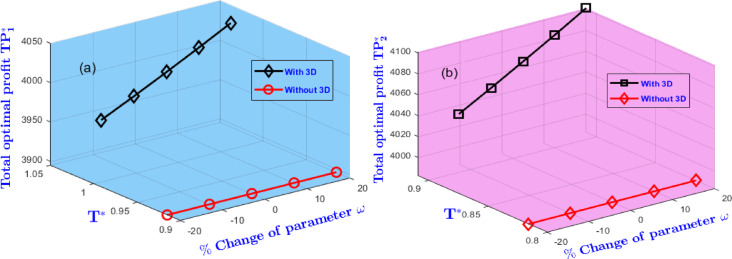
Sensitivity of total optimal profits by investing in AM technology to recycling rate (*ω*). (**a**) and (**b**) represents for with and without AM technology by ${TP}_{1}^{*}$ and ${TP}_{2}^{*}$ respectively.

According to Fig. 6, the comprehensive revenue of the inventory system exhibits a high sensitivity to the purchase cost ${C}_{p}$, and the total profit is lower. If the inventory system’s purchasing cost rises, the overall profit falls, and the cycle duration shortens slightly in two scenarios, as shown in Table 4. The supplier selection issue is essential since the overall profit is more vulnerable to the cost of purchases. The store could choose a supplier who can provide goods at a lesser cost and make more revenue. The overall profit is less sensitive to ordering and deterioration costs, as seen in the table. Another practical alternative is investing in technologies that reduce the expenses associated with ordering and degradation.

The overall profit is less sensitive to shortage costs $({C}_{b})$. Table 4 reflects that the total profit ${TP}_{1}^{*}$ is decreases with variations in time ${t}_{1}^{*}$ and ${T}_{1}^{*}$, displaying both increases and decreases. Concurrently, the total profit ${TP}_{2}^{*}$ exhibits a reduction with alternating increases and decreases in the time parameters ${t}_{2}^{*}$ and ${T}_{2}^{*}$. The impact of cost parameter sensitivity, specifically in relation to the investment in AM technology, results in a higher total profit compared to the sensitivity observed without such investment, as depicted in Fig. 5.

The sensitivity analysis in AM technology-related parameters from Fig. 6 the parameter u increases is the amount of recycled products after AM investment the total profit will be increased. The above Table 5 shows decreases in the amount of recycled products after AM investment; the total profit will be less. The plastic recycling sector has the potential to create high-quality recycled goods through the use of AM technology. This approach not only boosts the volume of recycled materials following investment in AM but also contributes to lowering emissions.

**Table 5:** Sensitivity analysis of parameters related to additive manufacturing technology on decision variables and objectives.

Parameter	$\%$	${t}_{1}^{*}\left(year\right)$	$\%{t}_{1}^{*}$	${T}_{1}^{*}\left(year\right)$	$\%{T}_{1}^{*}$	${TP}_{1}^{*}$	$\%{TP}_{1}^{*}$	${t}_{2}^{*}\left(year\right)$	$\%{t}_{2}^{*}$	${T}_{2}^{*} \left(year\right)$	$\%{T}_{2}^{*}$	${TP}_{2}^{*}\left({t}_{2}^{*}\right)$	$\%{TP}_{2}^{*}\left({t}_{2}^{*}\right)$
$A$	− 20	0.6202	− 0.43	1.0089	− 0.48	4000	− 0.10	0.5501	− 0.31	0.8876	− 0.41	4076	− 0.10
	− 10	0.6217	− 0.19	1.0116	− 0.22	4002	− 0.05	0.5510	− 0.14	0.8896	− 0.19	4078	− 0.05
	10	0.6240	0.18	1.0158	0.20	4005	0.02	0.5525	0.13	0.8927	0.16	4081	0.02
	20	0.6250	0.34	1.0174	0.36	4006	0.05	0.5531	0.24	0.8940	0.30	4082	0.05
$y$	− 20	0.5815	− 6.65	0.9394	− 7.34	3920	− 2.10	0.5248	− 4.89	0.8346	− 6.36	4005	− 1.84
	− 10	0.6011	− 3.50	0.9746	− 3.87	3961	− 1.07	0.5377	− 2.56	0.8616	− 3.33	4042	− 0.93
	10	0.6475	3.95	1.0579	4.35	40,481	1.10	0.5675	2.85	0.9240	3.67	4119	0.96
	20	0.6751	8.38	1.1078	9.27	4094	2.25	0.5848	5.98	0.9604	7.75	4160	1.96
$\omega$	− 20	0.6106	− 1.97	0.9917	− 2.18	3980	− 0.60	0.5439	− 1.43	0.8746	− 1.87	4059	− 0.51
	− 10	0.6167	− 1.00	1.0026	− 1.10	3992	− 0.30	0.5478	− 0.72	0.8829	− 0.94	4069	− 0.27
	10	0.6294	1.04	1.0254	1.14	4016	0.30	0.5560	0.76	0.8999	0.96	4091	0.27
	20	0.6360	2.10	1.0374	2.33	4028	0.60	0.5602	1.52	0.9088	1.96	4101	0.51
$e$	− 10	0.6218	− 0.18	1.0116	− 0.22	4001	− 0.07	0.5511	− 0.13	0.8896	− 0.19	4078	− 0.05
	− 5	0.6224	− 0.08	1.0127	− 0.11	4003	− 0.02	0.5514	0.07	0.8904	0.10	4082	− 0.02
	5	0.6235	0.10	1.0148	0.10	4005	0.02	0.5522	0.07	0.8920	0.08	4081	0.02
	10	0.6240	0.18	1.0517	0.19	4006	0.05	0.5525	0.13	0.8927	0.16	4082	0.05

**Fig.7:**
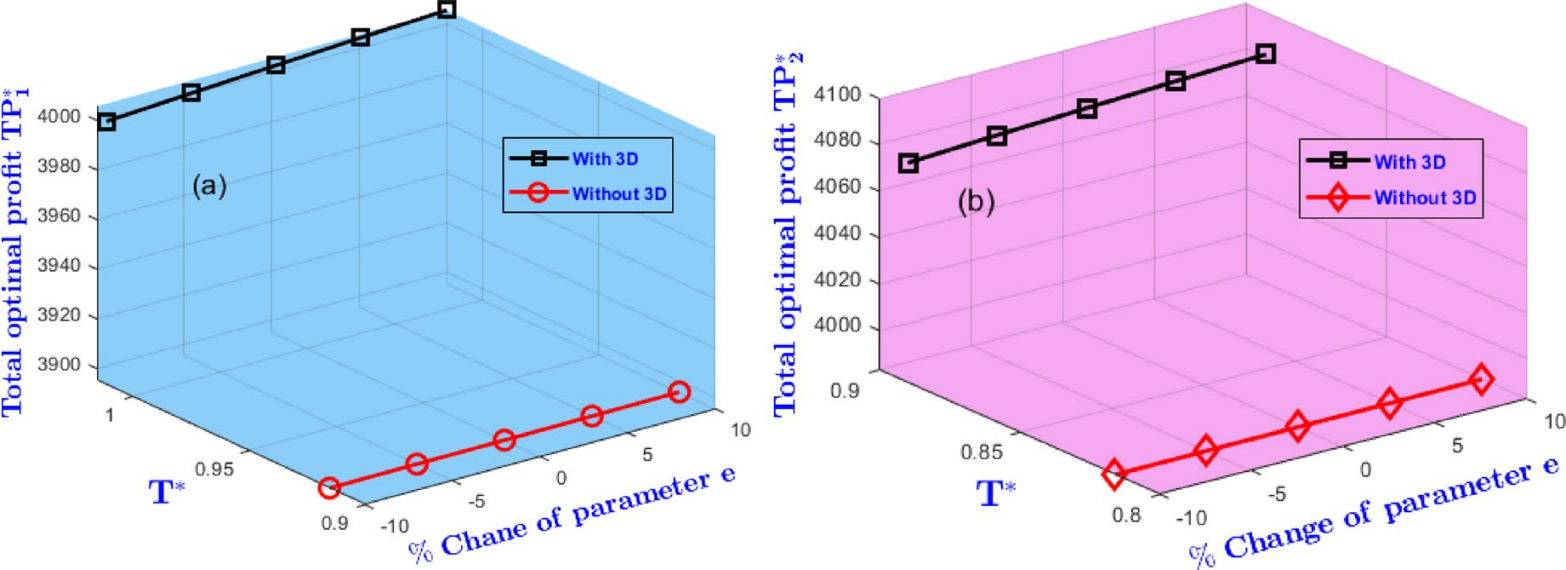
(**a**) and (**b**) illustrate the investment efficiency for with and without investment of AM technology for ${TP}_{1}^{*}$ and ${TP}_{2}^{*}$, respectively.

Increases the efficiency of the AM technology e the total profit will be increased. The above Table 4 shows the decreases in the total efficiency of the AM technology; the total profit will decrease. Sensitivity of the efficiency of AM technology parameter is shows in Fig. 7 for total optimal profits. Increasing efficiency can help the plastic reforming industry increase profits. Using AM technology, recycled plastic objects can be manufactured with higher quality.

**Fig. 8:**
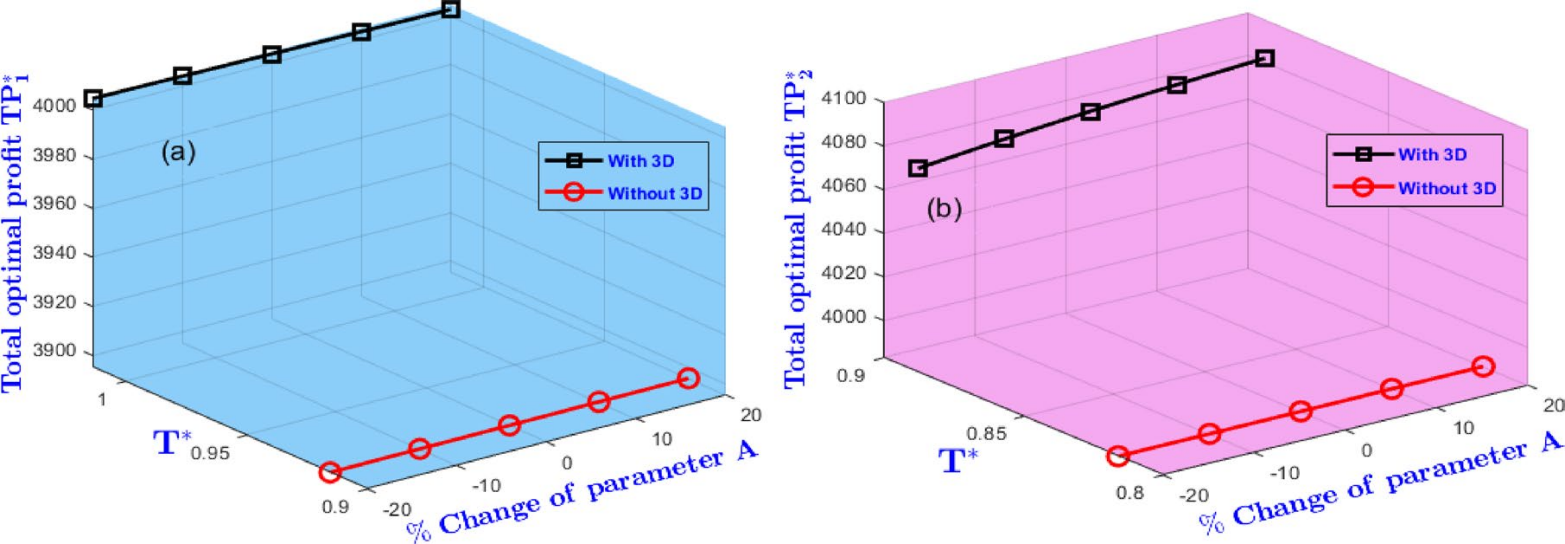
Impact of the AM technology investment parameter comparing investment scenarios on AM technology for ${TP}_{1}^{*}$ and ${TP}_{2}^{*}$ by (**a**) and (**b**), respectively.

Increases the investment of the AM technology automatically increases the quality of items in the inventory production system, and it will obtain high profit. The investment parameter A decreases automatically, the quality of the item will decrease, and the profit will decrease. Therefore, the plastic reforming company utilises AM technology to improve product quality and boost industrial profits. The graph illustrates the overall optimal profit for both cases in Fig. 8.

**Fig. 9:**
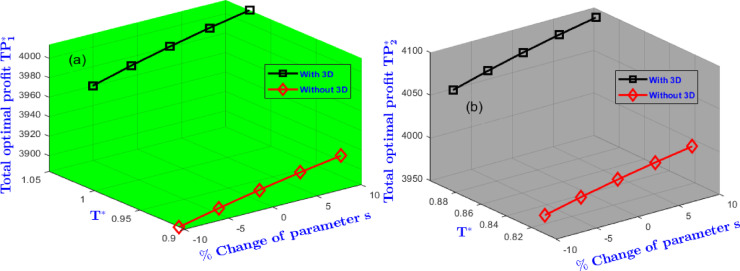
Effect of reliability parameter for with and without AM technology investment for ${TP}_{1}^{*}$ and ${TP}_{2}^{*}$ illustrating by (**a**) and (**b**), respectively.

Increases the supplier’s quality of the products s the total profit will be increase. Increasing the suppliers’ quality products increases the total profit correspondingly, increasing the product’s reliability $r(s)$ because product reliability depends on product quality. In Fig. 9 above, the total profit is increasing.

The sensitivity of the parameter p is high, i.e., the retailer selling the products at a high cost gets less profit. Hence at the time ${t}^{*}$ and ${T}^{*}$ are decreasing meanwhile, the optimal cost ${TP}_{1}^{*}$ and ${TP}_{2}^{*}$ are increasing as illustrates in Table 6. The demand for goods is mostly determined by the size of the market; hence stronger demand will be observed by retailers with a larger market share. More money will be made from the sale of goods when there is greater demand, as shown by the sensitivity of the parameter in Table 6. A decrease in profitability is expected when the pricing parameter is raised, as this will lead to a decline in item demand, resulting in lower profits from sales. To counteract this, enterprises can strategically employ promotional activities to expand their market share. This augmentation in market scope is expected to generate heightened demand, consequently increasing profitability.

**Table 6:** Sensitivity analysis of the different parameters incorporating AM investment by evaluating % of change of decision variables and objectives.

Parameter	$\%$	${t}_{1}^{*}\left(year\right)$	$\%{t}_{1}^{*}$	${T}_{1}^{*}\left(year\right)$	$\%{T}_{1}^{*}$	${TP}_{1}^{*}$	$\%{TP}_{1}^{*}$	${t}_{2}^{*}\left(year\right)$	$\%{t}_{2}^{*}$	${T}_{2}^{*} \left(year\right)$	$\%{T}_{2}^{*}$	${TP}_{2}^{*}\left({t}_{2}^{*}\right)$	$\%{TP}_{2}^{*}\left({t}_{2}^{*}\right)$
$a$	− 20	0.6486	4.13	1.0599	4.55	3045	− 23.95	0.5770	4.57	0.9495	6.63	3100	− 24.02
	− 10	0.6353	1.99	1.0360	2.19	3524	− 11.99	0.5640	2.21	0.9192	3.13	3589	− 12.03
	10	0.6115	− 1.83	0.9933	− 2.02	4484	11.99	0.5406	− 2.03	0.8656	− 2.88	4571	12.03
	20	0.6009	− 3.53	0.9742	− 3.91	4964	23.98	0.5301	− 3.93	0.8418	− 5.55	5064	24.12
$b$	− 5	0.6469	3.85	1.0569	4.25	4047	1.07	0.5671	2.77	0.9233	3.59	4119	0.96
	− 3	0.6370	2.26	1.0390	2.49	4029	0.62	0.5609	1.65	0.9101	2.11	4103	0.56
	10	0.5822	− 6.53	0.9408	− 7.20	3922	− 2.05	0.5252	− 4.82	0.8357	− 6.24	2006	− 1.81
	20	0.5489	− 11.88	0.8811	− 13.09	3845	− 3.97	0.5029	− 8.86	0.7891	− 11.47	3937	− 3.50
$x$	− 20	0.6107	− 1.96	0.9917	− 2.18	3980	− 0.60	0.5439	− 1.43	0.8746	− 1.87	4059	− 0.51
	− 10	0.6167	− 1.00	1.0026	− 1.10	3992	− 0.30	0.5478	− 0.72	0.8828	− 0.95	4069	− 0.27
	10	0.6294	1.04	1.0254	2.33	4016	0.30	0.5560	0.76	0.8999	0.96	4091	0.27
	20	0.6361	2.12	1.0374	2.33	4028	0.60	0.5602	1.52	0.9088	1.06	4101	0.51
$p$	− 5	0.6590	5.80	1.0787	6.40	3716	− 7.19	0.5744	4.108	0.9392	5.37	3786	− 7.21
	− 3	0.6443	3.44	1.0522	3.79	3832	− 4.30	0.5652	2.43	0.9197	3.19	3904	− 4.31
	10	0.5588	− 10.29	0.8990	− 20.81	4571	14.16	0.5105	− 7.48	0.8032	− 9.88	4666	14.24
	20	0.5049	− 18.94	0.8028	− 20.81	5132	28.17	0.4745	− 14.01	0.7264	− 18.50	5235	28.31
$s$	− 10	0.6153	− 1.22	1.0002	− 1.16	3989	− 0.37	0.5470	− 6.87	0.8810	− 1.16	4067	− 0.32
	− 5	0.6193	− 0.58	1.0074	− 0.63	3997	− 0.17	0.5495	− 0.42	0.8864	− 0.55	4074	− 0.15
	5	0.6262	0.53	1.0196	0.57	4010	0.15	1.5539	0.38	0.8955	0.47	4085	0.12
	10	0.6291	1	1.0248	1.09	4015	0.27	0.5558	0.72	0.8994	0.91	4090	0.25

## Management implications

This section presents key insights derived from the study, offering practical directions for managers involved in inventory planning and decision-making, particularly concerning sustainable management of recycled products.

- Efficient investment in AM technology enhances product quality and overall profitability. Adoption of advanced technological solutions not only strengthens manufacturing capability but also supports environmentally sustainable practices.
- Managers should maintain a balance between pricing and demand to sustain profitability. Excessively high prices may suppress demand; hence, competitive pricing combined with focused promotional strategies can stimulate sales. Furthermore, optimizing order quantities and inventory cycles enhances operational efficiency and long-term financial performance.
- Improving product quality enhances reliability, contributing to higher profitability. Manufacturers should prioritize robust quality control and continuous process improvement to ensure consistent performance, operational efficiency, and customer satisfaction.

## Conclusion

An eco-friendly inventory model has been developed for selling recycled plastic products sourced from the reprocessing industry. This model integrates an investment strategy in AM technology to enhance product quality, reliability, and overall profitability. By adopting 3D printing technology, this study demonstrates a reduction in emissions during production while simultaneously improving product quality. The proposed inventory system accounts for the deterioration rate of products, allowing businesses to optimise inventory levels, minimise costs, and increase profitability. A potential solution to financial constraints is the implementation of delayed payment strategies between suppliers and retailers. Table 2 presents the optimal total profit associated with varying delay payments in conjunction with investments in 3D printing, evaluated through a case study of a retailer’s business model to ensure practical applicability. As a result of integrating 3D printer investment, the total profit increases. When the payment delay is shorter than the cycle time, there is a 2.77% increase, and when the cycle time is less than the payment delay, the increase is 2.45%. To develop a sustainability model, elevate the quality standards of ecologically recycled products. The developed model determined the ideal values for the replenishment time and cycle time, thereby maximizing total profits.

Future research may extend the proposed EOQ-based framework by relaxing several simplifying assumptions adopted in the present study. The current model assumes constant deterioration and backordering parameters for analytical convenience. However, in practical retail environments, the deterioration of recycled plastic products may vary with storage duration, handling conditions, and environmental exposure. Therefore, future studies may incorporate time-dependent deterioration rates to capture the dynamic nature of product degradation during the inventory cycle. Similarly, customer willingness to accept backorders is often influenced by expected waiting time, suggesting that variable back ordering behaviour could provide a more realistic representation of demand fulfilment during shortage periods. Although the present model focuses on a single recycled product, AM systems are capable of producing multiple product variants using shared equipment. Extending the model to a multi-product inventory system would require capacity allocation, scheduling considerations, and potential competition among products for the same additive manufacturing resources.

In addition, environmental considerations may be integrated into the EOQ framework through carbon cap-and-trade regulations, which would allow emission-related costs or constraints to be incorporated into inventory decisions. It may also explore the integration of digital manufacturing technologies, such as generative artificial intelligence and digital twin platforms, to support adaptive decision-making and improve efficiency in sustainability.

## Data Availability

The datasets used and/or analysed during the current study available from the corresponding author on reasonable request.

## Parameters

$$s$$: Supplier’s quality products
$${I}_{e}$$: Annual interest rate earned
$${I}_{e}$$: Annual interest rate charged
$$D$$: Supplier-to-retailer-set credit period
$$\theta$$: Deterioration rate
$$p$$: The price at which the item is sold. ($/unit)
$$A$$: AM technology investment cost to increase quality ($/year)
$$e$$: Efficiency of the AM technology in recycling products
$$\omega$$: Amount of recycling products after 3d technology investment (ton/unit/year)
$$r$$: Reliability of the products
$$q$$: Fraction of quality items present in the inventory

## Decision variables

${t}_{1}$: The time at the reaching point of zero inventory level
$T$: Cycle length (unit of time)
